# The Dual-Identity Dilemma: A Comparative Study of Mentorship Structural Barriers in Military and Civilian Medical Education

**DOI:** 10.12688/mep.21548.1

**Published:** 2026-05-26

**Authors:** Zaw Phyo, Reshma Fatteh, Ye Phyo Aung, Manoj Kollukkad Mani, Sateesh Arja, Sireesha Bala. A, Tun Tun Naing, Tayzar Hein, Khin Aung Htun

**Affiliations:** 1 Medical Education Department, Defence Service Medical Academy (DSMA), yangon, 11021, Myanmar; 2 Avalon University School of Medicine (AUSOM), Willemst, Curaçaoad,, Willemstad, Curaçao, Netherlands Antilles

**Keywords:** Mentorship, Military Medical Education, International Collaboration, Professional Identity Formation, Dual-Identity Dilemma, Hierarchical Barriers, Mixed-Methods Research.

## Abstract

**Introduction:**

Mentorship functions as a stabilizing relational framework in high-demand medical education environments. This international collaborative study between the Defence Services Medical Academy (DSMA) and Avalon University School of Medicine (AUSOM) examines the “Dual-Identity Dilemma”—the structural tension between professional medical identity and institutional military role expectations. While mentorship is central to professional development, hierarchical military structures may shape its psychosocial accessibility.

**Methods:**

An explanatory sequential mixed-methods design (QUAN→QUAL) was conducted. Quantitative survey data were collected from 251 DSMA cadets and 100 AUSOM students to provide a civilian international benchmark. Within DSMA, qualitative interviews (n = 8 mentors) and focus groups (n = 8 mentees) explored identified quantitative “friction points.” Descriptive statistics, ANOVA with Bonferroni correction, and effect size reporting (partial η ^2^) were applied.

**Results:**

Mentorship expectations were high across both cohorts. However, DSMA cadets reported higher communication barriers (65.2%) compared with the civilian benchmark. Within DSMA, ANOVA revealed significant variation across academic years in communication barriers (p < .001, η ^2^p = .092) and career guidance (p = .001, η ^2^p = .084), with a pronounced Year 2 transitional peak. Qualitative findings identified hierarchical authority gradients as psychosocial barriers, while also revealing mentors’ role as “identity integrators” who model reconciliation between clinical professionalism and military discipline.

**Conclusion:**

Institutional structure fundamentally shapes in dual-role educational environments. The Dual-Identity Dilemma provides a conceptual lens for understanding how hierarchy moderates the depth of mentoring. Intentional, structurally responsive mentorship models are required to mitigate communication barriers and support identity integration in military medical education.

## I. Introduction

Mentorship is widely recognized as a cornerstone of medical education, supporting academic progression, professional identity formation, and psychosocial resilience ( El Tarhouny et al., 2026). In health professions education, effective mentoring relationships are associated with improved academic development, career planning, professional satisfaction, and sustained engagement, particularly when mentoring includes both career-oriented and psychosocial functions ( Burgess et al., 2018; Johnson & Griffin, 2024). Mentorship also supports self-efficacy and leadership development through role modelling, feedback, and reinforcement of competence beliefs ( Bandura & Walters, 1977). Contemporary scholarship further emphasizes that mentoring is most effective when it is intentionally structured, developmentally responsive, and supported by institutional faculty development systems ( Fountain & Newcomer, 2016; Zachary & Fain, 2022).

In high-demand training environments, mentorship functions not only as academic guidance but also as a stabilizing relational framework that promotes adaptive coping and professional growth ( Jones et al., 2011; Squires, 2019). Importantly, mentoring relationships depend on psychological safety—defined as the perception that one can ask questions, disclose uncertainty, and seek support without fear of embarrassment or punitive judgment ( Edmondson, 1999). When psychological safety is reduced, learners may avoid help-seeking and developmental conversations, limit the depth of psychosocial mentoring and weaken opportunities for growth and reflection ( Karabenick & Newman, 2013; Zachary & Fain, 2022).

Mentorship programs in public medical schools also benefit students’ personal and professional development. However, the comprehensive framework that ensures confidentiality should be considered to enhance its effectiveness ( Wasim et al., 2025). A great mentor is the one who provides opportunities and opens doors; reaches out to help students identify their strengths and achieve their goals; encourages them by serving as a positive example; and trains them in independent thinking ( Davis et al., 2023). A mentoring framework stands on pillars such as programmatic structure, oversight by a host organization, integration of mentoring with existing medical curricula, and recommendations for mentor and mentee training ( Tan et al., 2018). A framework for mentoring of medical students: thematic analysis of mentoring programmes between 2000 and 2015 by Tan et al. (2018) emphasized two key elements of an effective mentoring framework: flexibility and structure. Flexibility refers to meeting the needs of mentees and adapting to their changing needs. Structure concerns ensuring consistency in the mentoring process and compliance with prevailing codes of conduct and standards of practice ( Tan et al., 2018).

### Military medical education and structural complexity

In military medical education, mentorship becomes particularly complex because trainees must satisfy rigorous academic and clinical standards while simultaneously adhering to hierarchical military discipline ( Griffin, 2017; Johnson & Griffin, 2024). Authority gradients may constrain open mentor–mentee engagement and reduce the sense of safety required for candid communication—especially around stress, uncertainty, or perceived weakness ( Edmondson, 1999; Nembhard and Edmondson, 2006). Such dual-role structures may generate institutional tensions that affect communication accessibility, psychosocial safety, and professional identity formation ( McNeil et al., 2013). While civilian medical education also includes hierarchy, military settings may intensify its impact through rank, command structure, and expectations of mission-oriented obedience, creating distinctive mentoring barriers that warrant focused study.

**At Defence Services Medical Academy (DSMA), Myanmar,** the admissions procedure is characterized by extreme rigor, requiring candidates to pass competitive academic entrance tests alongside physical, teamwork, and psychometric assessments. Academic performance, clinical competency, and leadership development occur concurrently, producing substantial physical and psychological demands ( Moore II, 2024). The academy’s pedagogical philosophy is grounded in experiential learning and social learning, facilitating a transition toward student-centered approaches such as small group teaching ( Htun et al., 2024). In response to the pressures of this high-stakes environment, DSMA implemented a formal mentorship program in 2017 within an outcome-based integrated curriculum framework. Mentorship at DSMA therefore occurs within a dual-role educational ecosystem in which cadets must navigate the “Dual-Identity Dilemma,” functioning as both clinicians-in-training and disciplined military officers appointed as Medical Officers (Lieutenants) upon graduation.

Mentorship Avalon University School of Medicine (AUSOM) is structured primarily around academic support, residency (postgraduate) preparation, a 92% first-time applicants residency match success rate in 2025, and professional development within a non-military institutional culture. In the present study, AUSOM is used as a civilian international benchmark to descriptively contextualize mentorship functioning in a non-hierarchical, stable environment rather than as a causal control group.

### Conceptual framework: The Dual-Identity Dilemma

This study conceptualizes the Dual-Identity Dilemma as the structural tension experienced by military medical cadets who must simultaneously develop two competing professional identities: (1) a professional medical identity grounded in empathy, patient-centered care, ethical responsibility, and clinical autonomy; and (2) an institutional military identity grounded in discipline, hierarchy, rank, and mission-oriented obedience. Professional identity formation is increasingly recognized as a central outcome of medical education, shaping how learners internalize values, norms, and professional self-concept ( Wilson, 2024). In dual-role institutions, identity formation may be complicated by competing “they must become.”

Within this tension, mentorship may function as a moderating mechanism. Drawing on Kram’s ( Kram & Hall, 1989) distinction between career and psychosocial mentoring functions, mentorship may provide both practical professional guidance and identity-supportive role modelling. From a social learning perspective, mentors serve as models whose observed behaviors shape learners’ beliefs about competence and belonging ( Bandura & Walters, 1977). Where psychological safety is high, mentoring relationships can support open dialogue, reflective learning, and identity integration; where safety is constrained by hierarchy, mentoring may remain career-functional but become psychosocially shallow ( Edmondson, 1999; Nembhard and Edmondson, 2006; Zachary & Fain, 2022). Figure 1 illustrates the Dual-Identity Dilemma framework and the hypothesized moderating role of mentorship in supporting psychological safety, resilience, leadership development, and identity integration.

**Figure 1.**
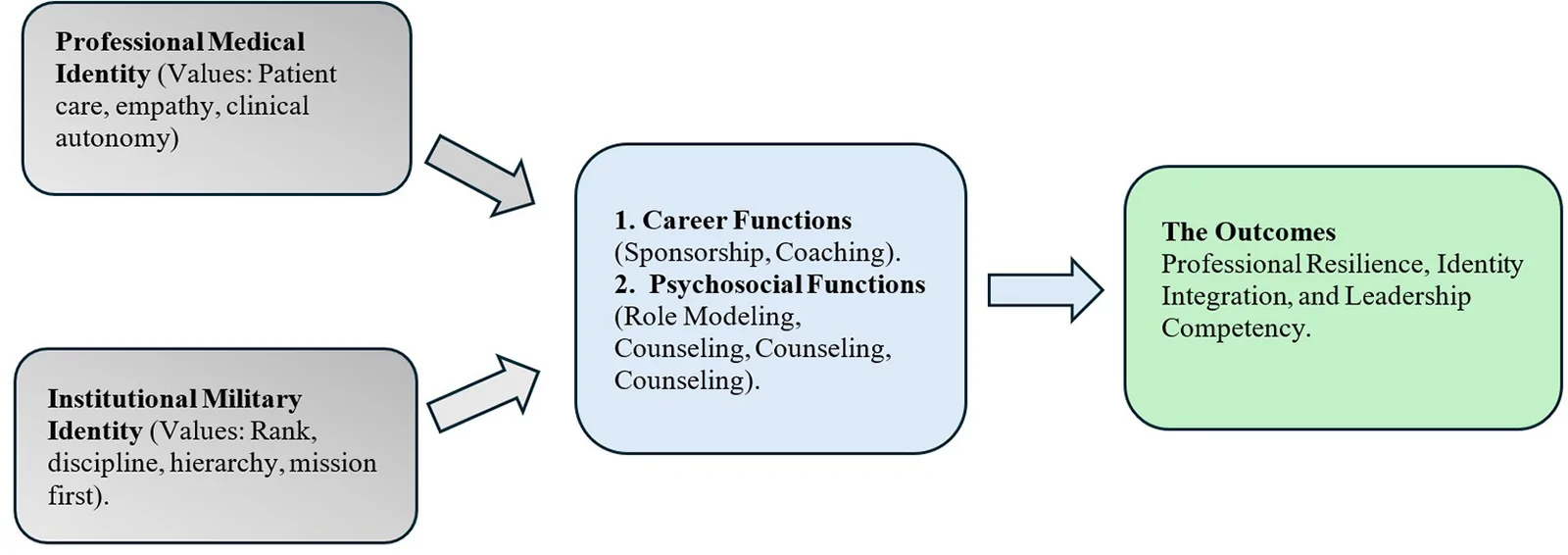
Conceptual framework of the Dual-Identity Dilemma. This model illustrates the structural tension between Professional Medical Identity (values: patient care, empathy, clinical autonomy) and Institutional Military Identity (values: rank, discipline, hierarchy, mission first). Mentorship serves as a moderating mechanism through Career Functions (sponsorship, coaching) and Psychosocial Functions (role modeling, counseling) to support outcomes of professional resilience, identity integration, and leadership competency.

This study, therefore, seeks to identify structural barriers to mentorship across military and civilian contexts and to inform mentorship design in dual-role educational environments.

### Study aims and research questions

This study, therefore, sought to identify structural barriers to mentorship across military and civilian contexts and to inform mentorship design in dual-role educational environments. The study addressed two research questions:

**RQ1:** How do mentorship perceptions differ between military and civilian medical education contexts?

**RQ2:** What structural barriers influence mentorship accessibility within a hierarchical military academy?

## II. Methods

### Research design

A comparative cross-sectional design was employed. Quantitative data were collected from both institutions to enable descriptive comparison. Within DSMA, an explanatory sequential mixed-methods approach (QUAN→QUAL) was applied ( Creswell & Clark, 2023; Ivankova, 2015). Qualitative inquiry was conducted exclusively within DSMA to explain quantitative “friction points” identified in the survey phase. AUSOM was included as a civilian international benchmark to contextualize mentorship functioning in a non-hierarchical institutional culture.

### Study setting and participants

At the Defence Services Medical Academy (DSMA) in Yangon, Myanmar, cadets undertake a six-year medical curriculum integrated with structured military training. Established in 1992 to address a critical scarcity of medical officers, the academy operates as a center of excellence with the primary mission of producing highly qualified medical professionals who possess high moral standards and the courage to serve the Myanmar Armed Forces. The admissions procedure is characterized by extreme rigor, requiring candidates to pass competitive academic entrance tests alongside physical, teamwork, and psychometric assessments.

In contrast, Avalon University School of Medicine (AUSOM) represents a civilian international medical education model that delivers a four-year MD program, and its continuous quality improvement is based on program surveys and students’ satisfaction ( Arja et al., 2025). AUSOM draws a diverse student body primarily from the United States, Canada, India, and Nigeria, focusing its curriculum on strong USMLE preparation and clinical rotations at teaching hospitals across the USA.

While the DSMA cohort operates under a military-medical mandate, the AUSOM cohort serves as a benchmark for international medical education standards. AUSOM holds dual accreditation from the Caribbean Accreditation Authority for Education in Medicine and other Health Professions (CAAM-HP) and the Accreditation Commission on Colleges of Medicine (ACCM). ACCM and CAAM-HP standards are comparable to those of the Liaison Committee on Medical Education (LCME), ensuring that its training is comparable to that of U.S. medical schools. These accreditation program surveys provide a baseline for comparing academic and other support systems in an environment where satisfaction is closely tied to academic and professional career support systems ( Arja et al., 2025).

### Instruments and data collection

Survey development was guided by Kram’s (1988) mentoring functions and Social Cognitive Theory ( Bandura & Walters, 1977). Domains included expectations, academic support, career guidance, personal development, communication barriers, mentor availability, and career influence. Content validity was established through expert review (CVI = .85; ICC = .82). Internal consistency was acceptable (Cronbach’s α = .79).

Quantitative data were collected at DMSA via paper-based surveys. Verbal consent was obtained at DMSA before administering the surveys. All students were invited from the foundation year through year 5. Participation in the survey was voluntary and anonymous. At AUSOM, the surveys were distributed through SurveyMonkey. All students who are actively enrolled in the pre-clinical program and clinical rotations (year 1–4) were invited. The surveys were open until sufficient responses were received. The participation in the survey was voluntary and anonymous. Since surveys were conducted through SurveyMonkey at AUSOM, students were informed that consenting to participate in this survey means they are consenting to be part of this study, and this was stated before beginning the survey. No personal information about students, including their names and student IDs, was identified through these surveys. The responses were compiled by AUSOM’s IT department.

### Qualitative phase (DSMA only)

A total of 10 interviews were conducted, including 8 mentor individual interviews and 2 focus groups (n = 8 mentees). Two focus groups were conducted, each with four mentees. The interviews were audio-recorded. Consent was taken before each interview. The interviews followed a semi-structured format. Even though interviews used a set of questionnaires, the participants were allowed to express their experiences and perceptions. Each interview lasted about 30–45 minutes.

### Data analysis

Quantitative analyses included descriptive statistics, one-way ANOVA (academic-year comparisons), and independent-samples t-tests (gender comparisons). Bonferroni correction was applied to ANOVA results (α = .0083) to reduce Type I error risk ( Sedgwick, 2012). Integration of findings was conducted through a joint display approach consistent with mixed-methods principles ( Guetterman et al., 2015).

Interviews were transcribed verbatim and analyzed using a step-by-step thematic analysis framework. MAXQDA software was used to transcribe the text. The investigators used thematic analysis because it is a suitable method for understanding experiences, perceptions, and opinions ( Kiger & Varpio, 2020; Braun & Clarke, 2012). The thematic analysis included familiarization, generating initial codes, searching for themes, reviewing, defining, naming themes, and writing the report ( Kiger & Varpio, 2020).

### Ethical considerations

The study was conducted in strict accordance with the ethical guidelines of the Defence Services Medical Academy (DSMA) Ethical and Research Committee and was formally approved under reference number DSMA/ERB07/2025/C-006. This research also received formal approval from the Research and Ethics Committee of Avalon University School of Medicine (AUSOM) under approval number AUSOM Ethics Ref: 001/4/2025. Participation was entirely voluntary and anonymous for all cohorts. Informed verbal consent was obtained from all participants at DSMA prior to both quantitative and qualitative data collection. Participants at AUSOM provided written informed consent via an electronic disclosure statement preceding the online survey.

### Informed consent

Before conducting the study, informed verbal consent was obtained from respondents to participate in the study at DMSA, prior to the collection of quantitative and qualitative data. Written consent was attached to the survey at AUSOM. Consenting to complete the survey means consenting to participate in a voluntary study.

## III. Results

### Comparative descriptive findings across institutions

DSMA Response Rate: A total of 251 undergraduate cadets participated (98.4% response rate). Participants represented Foundation Year through Year 5. Twenty-eight participants were female cadets from the first cohort of female admissions.

AUSOM Response Rate: The comparator cohort included 100 responses out of 200 (50% response rate) from actively enrolled MD program participants in the pre-clinical program and clinical rotations (years 1–4).

Mentorship was perceived as highly valuable in both institutional contexts; however, notable differences emerged between the civilian international benchmark (AUSOM) and the military academy setting (DSMA), particularly in satisfaction levels and communication accessibility. Because institutional contexts and response formats were not fully equivalent, cross-institutional comparisons are presented descriptively, with AUSOM used as a contextual benchmark rather than a causal control group.

Across domains, AUSOM students reported higher endorsement of mentorship expectations and satisfaction. At AUSOM, 95.0% of students reported high expectations for mentorship, compared with 80.4% of DSMA cadets (Mean = 4.0). Satisfaction with academic support was also higher at AUSOM (88.9%) than at DSMA (78.9%; Mean = 3.9), with a similar pattern for career guidance (88.9% vs. 79.5%; Mean = 4.0).

The clearest divergence involved communication accessibility. Whereas most AUSOM students reported communication as not a challenge, 65.2% of DSMA cadets reported moderate-to-significant communication barriers. This pattern suggests that institutional hierarchy may operate as a structural moderator of mentorship openness and approachability in military training environments ( Zachary & Fain, 2022) (see Table 1).

**Table 1.** Comparative mentorship perceptions across institutions (descriptive).

Mentorship domain	AUSOM Mean (SD)*	DSMA Mean (SD)
High Expectations	4.6 (0.4)	4.0 (0.7)
Academic Support Satisfaction	4.4 (0.5)	3.9 (0.8)
Career Guidance Satisfaction	4.4 (0.5)	4.0 (0.7)
Communication Barriers	1.8 (0.6)	3.2 (1.1)
Leadership Skill Development	3.7 (0.8)	3.7 (0.9)

*Note:* DSMA mean scores reflect Likert-scale responses (1 = strongly disagree, 5 = strongly agree). AUSOM’s mean scores were derived from equivalent Likert-scale survey items administered within the civilian cohort and are presented descriptively as institutional benchmark data. Direct statistical comparisons were not performed due to contextual and measurement differences between institutions.

Despite these contrasts, perceived leadership skill development was identical across institutions (72.6%), indicating that mentorship may retain developmental value even when psychosocial accessibility is structurally constrained.

### Variation across academic years within DSMA

To examine whether mentorship perceptions differed across stages of training within DSMA, one-way ANOVA was conducted across academic years.

Statistically significant differences were identified for:

• Career guidance: F(5, 245) = 4.49, p = .001, partial η ^2^ = .084
• Communication barriers: F(5, 245) = 4.97, p < .001, partial η ^2^ = .092

These effect sizes indicate moderate practical differences across training years, suggesting that mentorship accessibility and perceived career support vary meaningfully across the developmental trajectory.

As illustrated in Figure 2, a statistically significant divergence in mentorship perceptions occurs at the Year 2 transition, characterized by a sharp rise in communication barriers (p < .001) and the beginning of a downward trend in career guidance satisfaction (p = .001) (see Table 2).

**Figure 2.**
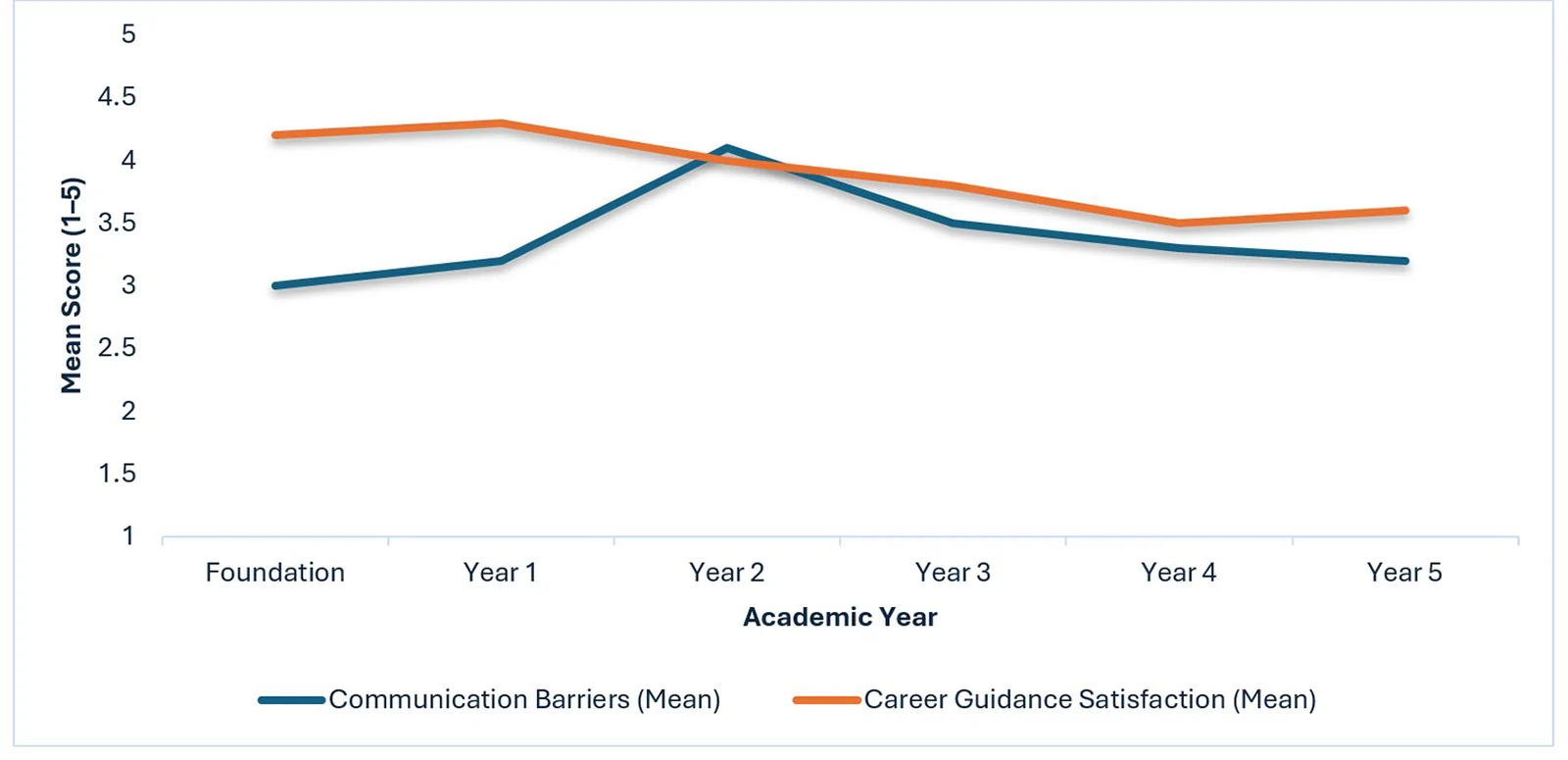
Longitudinal variation in mentorship perceptions across academic years at DSMA. The chart displays the shifts in mentorship domains from Foundation Year through Year 5. The intersection at Year 2 represents the critical phase of the Dual-Identity Dilemma, characterized by a statistically significant peak in communication barriers (74.0%) and the onset of declining satisfaction in career guidance.

**Table 2.** ANOVA results across academic years at DSMA (Including effect sizes).

Mentorship domain	F (5, 245)	p-value	Partial η ^2^	Significant After Bonferroni (α = .0083)
Career Guidance	4.49	.001	.084	Yes
Communication Barriers	4.97	< .001	.092	Yes
Balancing Medical & Military Roles	3.16	.009	.061	No
Academic Support	1.65	.148	.033	No

Partial η ^2^ values between .06 and .10 indicate moderate educationally meaningful differences, suggesting that mentorship perceptions shift in practically relevant ways across training stages rather than reflecting trivial statistical variation.

Career guidance satisfaction was highest in Year 1 and Year 2 and declined by Year 4, suggesting reduced perceived career support in later stages of training. Communication barriers peaked in Year 2 (74.0%), consistent with heightened transitional strain during early integration of military and medical responsibilities.

After applying Bonferroni correction (α = .0083), balancing medical and military responsibilities (Mean = 3.3; 66.1%) did not meet the adjusted significance threshold (p = .009), indicating that dual-role strain is a pervasive structural challenge rather than a developmentally specific phenomenon.

These findings suggest that while career guidance and communication accessibility vary moderately across academic stages, the challenge of balancing dual-role responsibilities remains consistently elevated throughout military medical training.

### Gender findings (exploratory)

Independent samples t-tests were conducted to explore gender differences within the DSMA cohort. A small statistically significant difference was observed in balancing medical and military responsibilities (p = .032), with male cadets reporting slightly higher perceived strain. No statistically significant gender differences were identified for academic support, career guidance, communication barriers, mentor availability, or career influence.

Given the small female subsample (n = 28), these findings should be interpreted as exploratory and hypothesis-generating (see Table 3).

**Table 3.** Gender comparison of key mentorship domains (DSMA).

Category	Male Mean (SD) (n=223)	Female Mean (SD) (n=28)	p-value
Academic support	3.96 (0.75)	3.79 (0.50)	.115
Career guidance	3.97 (0.68)	4.00 (0.27)	.660
Communication barriers	3.27 (1.10)	3.14 (0.89)	.478
Balancing dual roles	3.34 (0.91)	3.07 (0.90)	.032*

*Note:* p < .05 interpreted as exploratory; not Bonferroni adjusted.

### Qualitative findings (DSMA only)

Thematic analysis of mentor interviews and cadet focus groups produced three themes that contextualized quantitative “friction points.”

### Hierarchical barriers

Cadets and mentors described rank differentials and authority gradients as inhibiting open communication. Hesitancy in initiating contact—linked to hierarchical distance and generational gaps—aligned with elevated quantitative communication barrier scores.

Participants emphasized how the uniform creates a psychological wall. One cadet noted: “I want to ask my mentor about feeling overwhelmed, but he is a Colonel. I am afraid he will see my struggle as a lack of military discipline rather than a medical student’s stress.” This illustrates reluctance to engage in psychosocial mentoring due to rank differentials.

This qualitative evidence explains the quantitative ‘friction point’ seen in the survey data, where rank acts as a persistent psychosocial moderator that limits mentee outreach.

### Dual-role strain

Cadets described the concurrent demands of medical coursework and military training as emotionally taxing and cognitively demanding. This theme was reported across academic years, supporting the interpretation that dual-role strain is systemic.

### Mentorship as identity integration

Mentors were perceived not only as academic advisors but also as exemplars of successful dual-role reconciliation. Cadets described mentors as symbolic bridges between clinical competence and military professionalism, helping explain why leadership development remained robust despite structural constraints.

Mentors acknowledged this bridging role. One mentor stated, “I show them that my stethoscope and my rank insignia are not in conflict; one informs the other. You cannot be a good military doctor if you forget how to be a doctor first.”

### Integrated interpretation

Integrated interpretation indicates that institutional hierarchy shapes mentorship accessibility—particularly psychosocial openness—while mentorship benefits related to career guidance and leadership remain comparatively robust. Together, the findings support the Dual-Identity Dilemma as a structural condition in military medical education, in which mentorship outcomes are moderated by authority gradients and competing role expectations ( Kram, 1988; Zachary & Fain, 2022).

## IV. Discussion

This study demonstrates that mentorship is highly valued across both civilian and military medical education contexts; however, institutional structure shapes mentorship accessibility, particularly in the psychosocial domain. Using **AUSOM** as a civilian benchmark, **DSMA** cadets reported comparatively greater communication barriers and persistent strain associated with simultaneously meeting academic and military demands. The “Mentorship Paradox” identified at DSMA—characterized by high communication barriers (65.2%) despite high perceived value—contrasts sharply with the civilian model observed at Avalon. At AUSOM, where communication barriers were reported as minimal, the mentoring relationship is heavily focused on academic support and residency matching—an objective evidenced by their 92% first-time applicants’ residency (postgraduation placement) match success rate in 2025. Furthermore, while satisfaction in civilian contexts like Avalon is often driven by institutional success markers such as program surveys required by accreditation agencies, quality improvements, and student satisfaction rates ( Arja et al., 2025), the military context at DSMA necessitates a mentorship model focused on role reconciliation and resilience amid hierarchical dual-role strain.

### The Dual-Identity Dilemma as a structural condition

The Dual-Identity Dilemma represents tension between professional identity formation and institutional role conformity. In civilian contexts, mentorship appears primarily organized around academic coaching and career progression. In contrast, within military settings, mentorship must operate within hierarchical structures that may limit relational openness and constrain psychologically safe dialogue ( Griffin, 2017; Zachary & Fain, 2022).

The stark contrast with the civilian benchmark (AUSOM), where communication was reported as non-challenging, suggests that the barriers identified at DSMA may be influenced by structural characteristics of the military hierarchy rather than by medical education alone. This finding is reinforced by the observation that civilian students often focus on institutional success markers, such as success accreditation site visits, students’ satisfaction rates for educational program and student support systems, and continuous quality improvement ( Arja et al., 2025), whereas military cadets must navigate a much more rigid internal social structure. This structural friction serves as the foundation for the “Paradox of Resilience,” where cadets utilize a “Strategic Adaptation Toolkit” to transform this dual-identity conflict into professional growth ( Phyo et al., 2025). Structural consistency and flexibility with mentees are well recognized in literature as key elements of the frameworks involving mentor-mentee relationships ( Tan et al., 2018).

Consistent with Social Learning Theory, developmental confidence is shaped by accessible role modeling and supportive interactions ( Bandura & Walters, 1977). When authority gradients discourage help-seeking or candid disclosure, mentees may experience reduced confidence in initiating developmental conversations. In this way, hierarchy operates not merely as an administrative feature but as a psychosocial moderator of mentorship effectiveness. Thus, the Dual-Identity Dilemma extends beyond competing tasks and reflects a deeper tension between competing professional selves—being socialized simultaneously into the values of a compassionate healer and a disciplined military officer. In this high-stakes environment, the mentor must act as an “identity integrator,” demonstrating that reconciling these dual roles is not only a practical requirement but also a hallmark of professional excellence ( Phyo et al., 2025).

### Mentoring functions under the hierarchy

Kram’s (1988) framework distinguishes career functions (e.g., sponsorship, coaching) from psychosocial functions (e.g., counselling, affirmation). In DSMA, career guidance remained relatively strong overall, yet communication barriers suggest that psychosocial mentoring may be more difficult to access when relational approachability is constrained. This aligns with evidence that psychological safety is a key prerequisite for effective mentoring relationships ( Zachary & Fain, 2022).

### Developmental timing across academic years

Within DSMA, perceptions were not uniform across academic years. The decline in career guidance ratings by Year 4 suggests mentorship needs intensify as cadets approach major professional transitions. Communication barriers peaking in Year 2 may reflect a critical period of adjustment during which dual expectations become more salient. These findings support the need for stage-sensitive mentorship structures and structured support during transition points ( Pitts, 2009).

### Mentorship as identity integration

Qualitative findings extend the quantitative results by indicating that mentors may function as “identity integrators”—models of how clinical professionalism can coexist with military expectations. This integrative function may help explain why leadership development remained robust despite structural barriers. In dual-role institutions, mentorship may therefore serve not only as academic support but as a mechanism of identity reconciliation ( Kram & Hall, 1989).

By functioning as identity integrators, mentors provide observational learning, central to Social Learning Theory, demonstrating that the reconciliation of dual identities is achievable and may serve as a marker of excellence in this setting ( Bandura & Walters, 1977). This process is fundamental to resolving what has been termed the “Paradox of Resilience,” where the mentor facilitates the use of a “Strategic Adaptation Toolkit” to navigate the conflicting cultures of medicine and the military ( Phyo et al., 2025).

While the civilian model at AUSOM focuses on a singular professional identity, the military mentor at DSMA must perform a “dual-modeling” role, demonstrating that the values of a “healer”—such as empathy and patient advocacy—can coexist within the rigid norms of an “officer” without compromising either identity. This demonstrates to mentees that reconciling dual identities is not only a practical requirement but also a hallmark of professional excellence in the military-medical setting ( Phyo et al., 2025). Furthermore, while civilian mentorship satisfaction often aligns with external accreditation milestones ( Arja et al., 2025), the military mentor serves as the primary internal vehicle for navigating this complex identity.

### Practical implications

The findings suggest that mentorship models in dual-role training environments cannot rely solely on conventional structures designed for civilian medical education. Although DSMA cadets reported substantial benefits (career guidance and leadership development), the most prominent barriers—communication difficulty and persistent dual-role strain—appear embedded in hierarchical constraints. Where mentoring is formally assigned, but psychologically constrained, psychosocial mentoring functions may be reduced ( Zachary & Fain, 2022).

Accordingly, DSMA may benefit from a military-adapted mentorship approach that increases relational accessibility without undermining institutional discipline. Based on Social Learning Theory, developmental confidence is strengthened through approachable role modelling and supportive interaction ( Bandura & Walters, 1977). As proposed in the Military Mentorship Adaptation Model (see Figure 3), a tiered structure allows for the formal preservation of military hierarchy while creating a ‘safe’ base for psychosocial development through near-peer interactions. Strategies aligned with the present findings include:

1. Near-peer mentoring (e.g., Year 5 → Year 2) to reduce approachability barriers while maintaining organizational structure.
2. Protected mentorship time to reduce “mentoring as extra duty” perceptions and improve availability.
3. Mentor development training emphasizing psychosocial mentoring skills (listening, feedback, trust-building) alongside academic coaching, consistent with Kram’s (1988) distinction between career and psychosocial functions.
4. Flexible communication pathways, including asynchronous options, to reduce authority-related barriers while preserving organizational norms ( Zachary & Fain, 2022).

**Figure 3.**
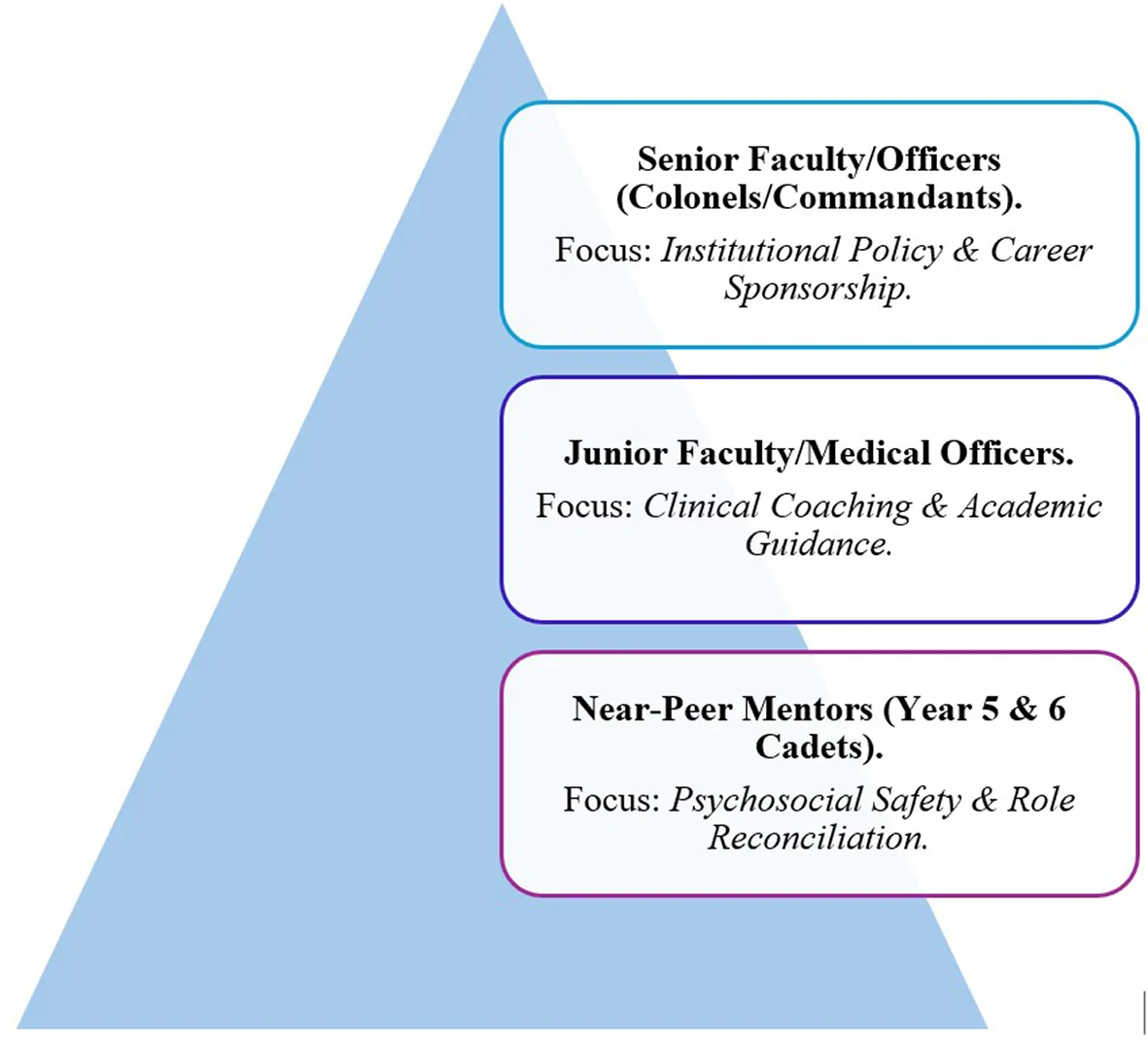
Military mentorship adaptation model (proposed). A tiered mentorship framework designed for dual-role educational environments. The model integrates near-peer mentoring (Year 5 students mentoring Year 2 students) to reduce authority-gradient barriers and improve relational accessibility. This system operates alongside senior faculty mentors and protected mentorship time to preserve military hierarchy while fostering a “safe base” for psychosocial development.

### Strengths and limitations

Strengths include the near-census DSMA response rate and the explanatory sequential mixed-methods design, which enabled qualitative contextualization of quantitative patterns ( Creswell & Clark, 2023). Limitations include descriptive (non-causal) cross-institutional comparison, absence of qualitative data from AUSOM, and limited female representation.

Several limitations should be considered when interpreting these findings. First, the comparison between DSMA and AUSOM is descriptive rather than causal. The institutions differ substantially in mission. These contextual differences introduce potential confounding variables that prevent attribution of observed differences solely to the military–civilian distinction. Accordingly, AUSOM is best interpreted as a contextual benchmark rather than an experimental control group.

Second, qualitative inquiry was conducted only at DSMA. Explanatory mechanisms (“why”) are therefore grounded in the military context alone, while civilian findings remain limited to survey description. Including qualitative inquiry in future research at AUSOM would strengthen comparative interpretation.

Third, although moderate effect sizes (partial η ^2^) were reported for key ANOVA findings, future studies should strengthen interpretation by reporting confidence intervals and additional measures of practical significance. In large samples, statistical significance may not reflect practical significance. Future reporting should include effect-size estimates (e.g., partial η ^2^) alongside ANOVA outcomes to enhance interpretability.

Fourth, the gender analysis is exploratory due to the small female subsample. Limited statistical power may obscure meaningful differences. As female cadet representation increases, future studies should examine whether mentorship experiences differ by gender, particularly with respect to psychological safety and identity negotiation.

Finally, although the manuscript conceptualizes a Dual-Identity Dilemma, quantitative measures primarily captured dual-role strain (balancing responsibilities) rather than identity-level conflict. While qualitative themes suggest identity integration through role modeling, future studies should include explicit identity formation measures and identity-focused qualitative prompts to strengthen construct alignment ( Bandura & Walters, 1977; Kram & Hall, 1989).

## V. Conclusion

Mentorship in military medical education operates within structural conditions that differ meaningfully from civilian contexts. While mentorship remains highly valued across both settings, hierarchical authority gradients and dual-role demands shape its accessibility and the depth of its psychosocial impact within military institutions.

This study conceptualizes the Dual-Identity Dilemma as a structural tension between professional medical identity and institutional military expectations, demonstrating that mentorship functions not only as academic guidance but also as a mechanism of identity integration. When intentionally designed, mentorship can mitigate communication barriers, support psychological safety, and promote resilient professional development.

Dual-role institutions must therefore move beyond conventional civilian mentorship models and adopt structurally responsive frameworks that acknowledge hierarchy while fostering relational accessibility. By implementing tiered, developmentally sensitive mentorship structures, military medical academies can better support identity reconciliation, leadership development, and sustainable professional formation. 

## Data Availability

The data supporting this study’s findings are openly available in the Figshare repository at: https://doi.org/10.6084/m9.figshare.31388209 ( Phyo et al., 2026). Phyo, Z., Aung, Y. P., & Fatteh, R. A. (2026). The Dual-Identity Dilemma: Mentorship Structural Barriers Dataset. *Figshare.* https://doi.org/10.6084/m9.figshare.31388209
• License Data are available under the terms of the Creative Commons Attribution 4.0 International license (CC-BY 4.0).
