# The Dual-Identity Dilemma: A Comparative Study of Mentorship Structural Barriers in Military and Civilian Medical Education

**DOI:** 10.12688/mep.21548.2

**Published:** 2026-09-12

**Authors:** Zaw Phyo, Reshma Fatteh, Ye Phyo Aung, Manoj Kollukkad Mani, Sateesh Arja, Sireesha Bala. A, Tun Tun Naing, Tayzar Hein, Khin Aung Htun

**Affiliations:** 1 Medical Education Department, Defence Service Medical Academy (DSMA), Yangon, Myanmar; 2 Avalon University School of Medicine (AUSOM), Willemstad, Curaçao, Netherlands Antilles

**Keywords:** Mentorship, Military Medical Education, International Collaboration, Professional Identity Formation, Dual-Identity Dilemma, Hierarchical Barriers, Mixed-Methods Research.

## Abstract

**Introduction:**

Mentorship functions as a stabilizing relational framework in high-demand medical education environments. This international collaborative study between the Defence Services Medical Academy (DSMA) and Avalon University School of Medicine (AUSOM) examines the “Dual-Identity Dilemma”—the structural tension between professional medical identity and institutional military role expectations. While mentorship is central to professional development, hierarchical military structures may shape its psychosocial accessibility.

**Methods:**

An explanatory sequential mixed-methods design (QUAN→QUAL) was conducted. Quantitative survey data were collected from 251 DSMA cadets and 100 AUSOM students to provide a civilian international benchmark. Within DSMA, qualitative interviews (n = 8 mentors) and focus groups (n = 8 mentees) explored identified quantitative “friction points.” Descriptive statistics, ANOVA with Bonferroni correction, and effect size reporting (partial η ^2^) were applied.

**Results:**

Mentorship expectations were high across both cohorts. However, DSMA cadets reported higher communication barriers (65.2%) compared with the civilian benchmark. Within DSMA, ANOVA revealed significant variation across academic years in communication barriers (p < .001, η ^2^p = .092) and career guidance (p = .001, η ^2^p = .084), with Year 2 showing the highest descriptive communication-barrier level. Qualitative findings identified perceived hierarchical authority gradients as barriers to psychosocial openness and suggested that mentors may function as ‘identity integrators’ by modelling ways in which clinical and military role expectations can coexist.

**Conclusion:**

Institutional context may influence mentorship accessibility in dual-role educational environments. The Dual-Identity Dilemma provides a conceptual lens for interpreting how hierarchical authority relationships and competing role expectations may affect psychosocial mentoring in military medical education. Structurally responsive mentorship approaches warrant further evaluation.

## I. Introduction

Mentorship is widely recognized as a cornerstone of medical education, supporting academic progression, professional identity formation, and psychosocial resilience ( El Tarhouny et al., 2026). In health professions education, effective mentoring relationships are associated with improved academic development, career planning, professional satisfaction, and sustained engagement, particularly when mentoring includes both career-oriented and psychosocial functions ( Burgess et al., 2018; Johnson & Griffin, 2024). Mentorship also supports self-efficacy and leadership development through role modelling, feedback, and reinforcement of competence beliefs ( Bandura & Walters, 1977). Contemporary scholarship further emphasizes that mentoring is most effective when it is intentionally structured, developmentally responsive, and supported by institutional faculty development systems ( Fountain & Newcomer, 2016; Zachary & Fain, 2022).

In high-demand training environments, mentorship functions not only as academic guidance but also as a stabilizing relational framework that promotes adaptive coping and professional growth ( Jones & Brown, 2011; Squires, 2019). Importantly, mentoring relationships depend on psychological safety—defined as the perception that one can ask questions, disclose uncertainty, and seek support without fear of embarrassment or punitive judgment ( Edmondson, 1999). When psychological safety is reduced, learners may avoid help-seeking and developmental conversations, limit the depth of psychosocial mentoring and weaken opportunities for growth and reflection ( Karabenick & Newman, 2013; Zachary & Fain, 2022).

Mentorship programs in public medical schools also benefit students’ personal and professional development. However, the comprehensive framework that ensures confidentiality should be considered to enhance its effectiveness ( Wasim et al., 2025). A great mentor is the one who provides opportunities and opens doors; reaches out to help students identify their strengths and achieve their goals; encourages them by serving as a positive example; and trains them in independent thinking ( Davis et al., 2023). A mentoring framework stands on pillars such as programmatic structure, oversight by a host organization, integration of mentoring with existing medical curricula, and recommendations for mentor and mentee training ( Tan et al., 2018). A framework for mentoring of medical students: thematic analysis of mentoring programmes between 2000 and 2015 by Tan et al. (2018) emphasized two key elements of an effective mentoring framework: flexibility and structure. Flexibility refers to meeting the needs of mentees and adapting to their changing needs. Structure concerns ensuring consistency in the mentoring process and compliance with prevailing codes of conduct and standards of practice ( Tan et al., 2018).

### Military medical education and structural complexity

In military medical education, mentorship becomes particularly complex because trainees must satisfy rigorous academic and clinical standards while simultaneously adhering to hierarchical military discipline ( Griffin, 2017; Johnson & Griffin, 2024). Authority gradients may constrain open mentor–mentee engagement and reduce the sense of safety required for candid communication—especially around stress, uncertainty, or perceived weakness ( Edmondson, 1999; Nembhard and Edmondson, 2006). Such dual-role structures may generate institutional tensions that affect communication accessibility, psychosocial safety, and professional identity formation ( McNeil et al., 2013). While civilian medical education also includes hierarchy, military settings may intensify its impact through rank, command structure, and expectations of mission-oriented obedience, creating distinctive mentoring barriers that warrant focused study.

**At Defence Services Medical Academy (DSMA), Myanmar,** the admissions procedure is characterized by extreme rigor, requiring candidates to pass competitive academic entrance tests alongside physical, teamwork, and psychometric assessments. Academic performance, clinical competency, and leadership development occur concurrently, producing substantial physical and psychological demands ( Moore II, 2024). The academy’s pedagogical philosophy is grounded in experiential learning and social learning, facilitating a transition toward student-centered approaches such as small group teaching ( Htun et al., 2024). In response to the pressures of this high-stakes environment, DSMA implemented a formal mentorship program in 2017 within its outcome-based integrated curriculum framework. Mentorship at DSMA therefore occurs within a dual-role educational ecosystem in which cadets must navigate the “Dual-Identity Dilemma,” functioning as both clinicians-in-training and disciplined military officers appointed as Medical Officers (Lieutenants) upon graduation.

Mentorship at Avalon University School of Medicine (AUSOM) is structured primarily around academic support, residency (postgraduate) preparation, a 92% first-time applicants residency match success rate in 2025, and professional development within a non-military institutional culture. In the present study, AUSOM is used as a civilian international benchmark to descriptively contextualize mentorship functioning within a different civilian medical education context rather than as a causal control group.

### Conceptual framework: The Dual-Identity Dilemma

This study conceptualizes the Dual-Identity Dilemma as the structural tension experienced by military medical cadets who must simultaneously develop two competing professional identities: (1) a professional medical identity grounded in empathy, patient-centered care, ethical responsibility, and clinical autonomy; and (2) an institutional military identity grounded in discipline, hierarchy, rank, and mission-oriented obedience. Professional identity formation is increasingly recognized as a central outcome of medical education, shaping how learners internalize values, norms, and professional self-concept ( Wilson, 2024). In dual-role institutions, identity formation may be complicated by potentially competing expectations regarding the professional roles that trainees are expected to assume.

Within this tension, mentorship may function as a supportive mechanism. Drawing on Kram’s (1988) distinction between career and psychosocial mentoring functions, mentorship may provide both practical professional guidance and identity-supportive role modelling. From a social learning perspective, mentors serve as models whose observed behaviors shape learners’ beliefs about competence and belonging ( Bandura & Walters, 1977). Where psychological safety is high, mentoring relationships can support open dialogue, reflective learning, and identity integration; where safety is constrained by hierarchy, mentoring may remain career-functional but become psychosocially shallow ( Edmondson, 1999; Nembhard and Edmondson, 2006; Zachary & Fain, 2022). Figure 1 illustrates the Dual-Identity Dilemma framework and the hypothesized supportive role of mentorship in supporting psychological safety, resilience, leadership development, and identity integration. Identity conflict was not directly measured quantitatively; identity-related interpretations are therefore hypothesis-generating.

**Figure 1.**
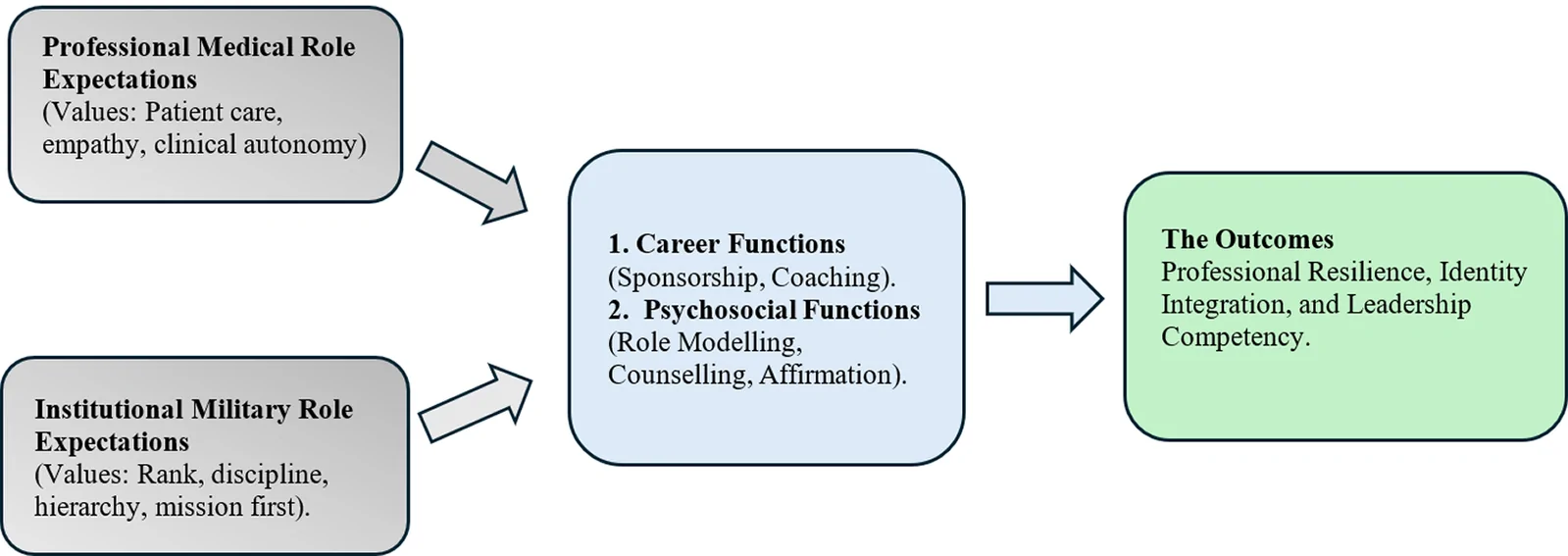
Conceptual framework of the Dual-Identity Dilemma. This hypothesis-generating model depicts proposed relationships between professional medical-role expectations, institutional military-role expectations, mentorship functions, and developmental outcomes. These proposed relationships were not directly tested as a structural or moderation model in the present study.

### Study aims and research questions

This study sought to examine mentorship perceptions across the two institutions, identify structural barriers within DSMA, and inform mentorship design in dual-role educational environments.

**RQ1:** How do mentorship perceptions differ between military and civilian medical education contexts?

**RQ2:** What structural barriers influence mentorship accessibility within a hierarchical military academy?

## II. Methods

### Research design

A comparative cross-sectional design was employed. Quantitative data were collected from both institutions to enable descriptive comparison. Within DSMA, an explanatory sequential mixed-methods approach (QUAN→QUAL) was applied ( Creswell & Clark, 2023; Ivankova, 2015). Qualitative inquiry was conducted exclusively within DSMA to explain quantitative “friction points” identified in the survey phase. AUSOM was included as a civilian international benchmark to contextualize mentorship functioning within a different civilian medical education context.

The DSMA quantitative and qualitative dataset analyzed in the present study has previously been reported in an institution-focused mixed-methods evaluation of the DSMA mentorship program ( Phyo et al., 2025a). The present study represents a distinct comparative and conceptual analysis in which the existing DSMA data are contextualized against an AUSOM civilian international benchmark and interpreted through the Dual-Identity Dilemma framework. No new DSMA participants were recruited for the present comparative analysis.

### Study setting and participants

At the Defence Services Medical Academy (DSMA) in Yangon, Myanmar, cadets undertake a six-year medical curriculum integrated with structured military training. Established in 1992 to address a critical scarcity of medical officers, the academy operates as a center of excellence with the primary mission of producing highly qualified medical professionals who possess high moral standards and the courage to serve the Myanmar Armed Forces. The admissions procedure is characterized by extreme rigor, requiring candidates to pass competitive academic entrance tests alongside physical, teamwork, and psychometric assessments.

In contrast, Avalon University School of Medicine (AUSOM) represents a civilian international medical education model that delivers a four-year MD program, and its continuous quality improvement is based on program surveys and students’ satisfaction ( Arja et al., 2025). AUSOM draws a diverse student body primarily from the United States, Canada, India, and Nigeria, focusing its curriculum on strong USMLE preparation and clinical rotations at teaching hospitals across the USA.

While the DSMA cohort operates under a military-medical mandate, the AUSOM cohort serves as a benchmark for international medical education standards. AUSOM holds dual accreditation from the Caribbean Accreditation Authority for Education in Medicine and other Health Professions (CAAM-HP) and the Accreditation Commission on Colleges of Medicine (ACCM). ACCM and CAAM-HP standards are comparable to those of the Liaison Committee on Medical Education (LCME), ensuring that its training is comparable to that of U.S. medical schools. These accreditation program surveys provide a baseline for comparing academic and other support systems in an environment where satisfaction is closely tied to academic and professional career support systems ( Arja et al., 2025).

### Instruments and data collection

Survey development was guided by Kram’s (1988) mentoring functions and Social Cognitive Theory ( Bandura & Walters, 1977). Domains included expectations, academic support, career guidance, personal development, communication barriers, mentor availability, and career influence. Content validity was established through expert review (CVI = .85; ICC = .82). Internal consistency was acceptable (Cronbach’s α = .79).

Quantitative data were collected at DSMA via paper-based surveys. Verbal consent was obtained at DSMA before administering the surveys. All students were invited from the foundation year through year 5. Participation in the survey was voluntary and anonymous. To reduce perceived coercion and authority-related response bias, DSMA surveys were distributed and collected by non-teaching staff, and completed questionnaires were returned in sealed envelopes. During instrument piloting, particular attention was given to clarity, culturally appropriate and rank-sensitive wording, and minimizing concerns related to social desirability and fear of reprisal.

At AUSOM, the surveys were distributed through SurveyMonkey. All students who are actively enrolled in the pre-clinical program and clinical rotations (year 1–4) were invited. The surveys were open until sufficient responses were received. The participation in the survey was voluntary and anonymous. Since surveys were conducted through SurveyMonkey at AUSOM, students were informed that consenting to participate in this survey means they are consenting to be part of this study, and this was stated before beginning the survey. No personal information about students, including their names and student IDs, was identified through these surveys. The responses were compiled by AUSOM’s IT department.

### Qualitative phase (DSMA only)

The qualitative phase used purposive sampling to obtain complementary mentor and mentee perspectives relevant to quantitative ‘friction points’ identified during Phase I. Sampling sought variation in academic year, mentoring experience, and participant perspective. Eight mentors participated in individual semi-structured interviews, while eight mentees participated across two focus groups, with four mentees in each group. Sessions were conducted in private settings by non-supervisory faculty, and interviewer–participant pairings avoided direct supervisory or hierarchical relationships to facilitate candid discussion and reduce perceived coercion. Sessions were conducted using a semi-structured guide, audio-recorded with consent, and transcribed verbatim. Each session lasted approximately 30–45 minutes. Qualitative data collection continued until thematic saturation was reached, operationalized as the point at which no substantively new themes or subthemes relevant to the explanatory research questions emerged. Field notes were maintained to document contextual observations and analytic reflections.

### Data analysis

Quantitative analyses were performed using IBM SPSS Statistics version 25. Descriptive statistics were used to summarize mentorship perception scores. One-way ANOVA was used for academic-year comparisons, and independent-samples t-tests were used for exploratory gender comparisons. Before parametric analysis, distributional assumptions were assessed using the Shapiro–Wilk test for normality and Levene’s test for homogeneity of variance. Where homogeneity of variance was not satisfied, Welch-adjusted analyses were used to assess the robustness of the findings. A Bonferroni-adjusted significance threshold (α = .0083) was applied to the academic-year domain analyses to reduce family-wise Type I error ( Sedgwick, 2012). Effect sizes were reported using partial η ^2^. Integration of quantitative and qualitative findings was conducted through a joint-display approach consistent with mixed-methods principles ( Guetterman et al., 2015).

Although responses were recorded using five-point Likert scales, parametric analyses were retained because t-tests and ANOVA are generally robust to common departures from interval-level measurement in Likert-type data ( Norman, 2010); distributional assumptions were additionally assessed using the Shapiro–Wilk and Levene tests, with Welch-adjusted analyses used where appropriate.

Qualitative data were analyzed thematically following Braun and Clarke’s approach ( Braun & Clarke, 2012). Coding proceeded iteratively using a shared codebook. Developing codes and themes were reviewed by the analytic team, with discrepancies discussed and resolved through consensus. Coding agreement was addressed through consensus-based analytic review rather than through calculation of a formal kappa coefficient. An audit trail was maintained to document coding and thematic decisions.

Trustworthiness was further supported through analyst triangulation, reflexive memoing, and examination of discrepant cases. MAXQDA was used to support organization and management of the qualitative data. Because members of the research team were institutionally affiliated with DSMA, researcher positionality was considered throughout analysis. Institutional familiarity provided contextual understanding but could also influence interpretation of authority-related experiences; reflexive memoing and analytic discussion were therefore used to identify and critically examine such assumptions during theme development ( Kiger & Varpio, 2020).

### Ethical considerations

The study was conducted in strict accordance with the ethical guidelines of the Defence Services Medical Academy (DSMA) Ethical and Research Committee and was formally approved under reference number DSMA/ERB07/2025/C-006. This research also received formal approval from the Research and Ethics Committee of Avalon University School of Medicine (AUSOM) under approval number AUSOM Ethics Ref: 001/4/2025. Participation was entirely voluntary and anonymous for all cohorts. Informed verbal consent was obtained from all participants at DSMA prior to both quantitative and qualitative data collection. Participants at AUSOM provided electronic informed consent through a disclosure statement presented before the online survey.

### Informed consent

At DSMA, informed verbal consent was obtained before participation in both the quantitative and qualitative components of the study. At AUSOM, electronic informed consent was obtained through a disclosure statement presented before the online survey. Participation in all study components was voluntary.

## III. Results

### Comparative descriptive findings across institutions

DSMA Response Rate: A total of 251 undergraduate cadets participated (98.4% response rate). Participants represented Foundation Year through Year 5. Twenty-eight participants were female cadets from the first cohort of female admissions.

AUSOM Response Rate: The comparator cohort included 100 responses out of 200 (50% response rate) from actively enrolled MD program participants in the pre-clinical program and clinical rotations (years 1–4).

Mentorship was perceived as highly valuable in both institutional contexts; however, notable differences emerged between the civilian international benchmark (AUSOM) and the military academy setting (DSMA), particularly in satisfaction levels and communication accessibility. Because institutional contexts and response formats were not fully equivalent, cross-institutional comparisons are presented descriptively, with AUSOM used as a contextual benchmark rather than a causal control group.

Across domains, AUSOM students reported higher endorsement of mentorship expectations and satisfaction. At AUSOM, 95.0% of students reported high expectations for mentorship, compared with 80.4% of DSMA cadets (Mean = 4.0). Satisfaction with academic support was also higher at AUSOM (88.9%) than at DSMA (78.9%; Mean = 3.9), with a similar pattern for career guidance (88.9% vs. 79.5%; Mean = 4.0).

The clearest divergence involved communication accessibility. Whereas most AUSOM students reported communication as not a challenge, 65.2% of DSMA cadets reported moderate-to-significant communication barriers. This descriptive pattern highlights differences in mentorship accessibility between the two institutional contexts. However, because DSMA and AUSOM differ in curriculum structure, national and institutional context, learner composition, survey administration, and response rate, the observed differences cannot be attributed specifically to military hierarchy. The potential influence of hierarchical authority relationships was explored more directly in the within-DSMA qualitative phase (see Table 1).

**Table 1.** Comparative mentorship perceptions across institutions (descriptive).

Mentorship domain	AUSOM Mean (SD) ^*^	DSMA Mean (SD)
High Expectations	4.6 (0.4)	4.0 (0.7)
Academic Support Satisfaction	4.4 (0.5)	3.9 (0.8)
Career Guidance Satisfaction	4.4 (0.5)	4.0 (0.7)
Communication Barriers	1.8 (0.6)	3.2 (1.1)
Leadership Skill Development	3.7 (0.8)	3.7 (0.9)

*Note:* DSMA mean scores reflect Likert-scale responses (1 = strongly disagree, 5 = strongly agree).

^*^ AUSOM’s mean scores were derived from equivalent Likert-scale survey items administered within the civilian cohort and are presented descriptively as institutional benchmark data. Direct statistical comparisons were not performed due to contextual and measurement differences between institutions.

### Variation across academic years within DSMA

To examine whether mentorship perceptions differed across stages of training within DSMA, one-way ANOVA was conducted across academic years.

Statistically significant differences were identified for:

• Career guidance: F(5, 245) = 4.49, p = .001, partial η ^2^ = .084
• Communication barriers: F(5, 245) = 4.97, p < .001, partial η ^2^ = .092

Before parametric analysis, assumption checks identified no severe departures from normality. Where Levene's test identified heterogeneity, Welch-adjusted analyses yielded the same substantive conclusions.

The overall academic-year effects were statistically significant for communication barriers (p < .001) and career guidance (p  = .001). Descriptively, Year 2 respondents showed the highest communication-barrier level (74.0%) (see Table 2).

**Figure 2.**
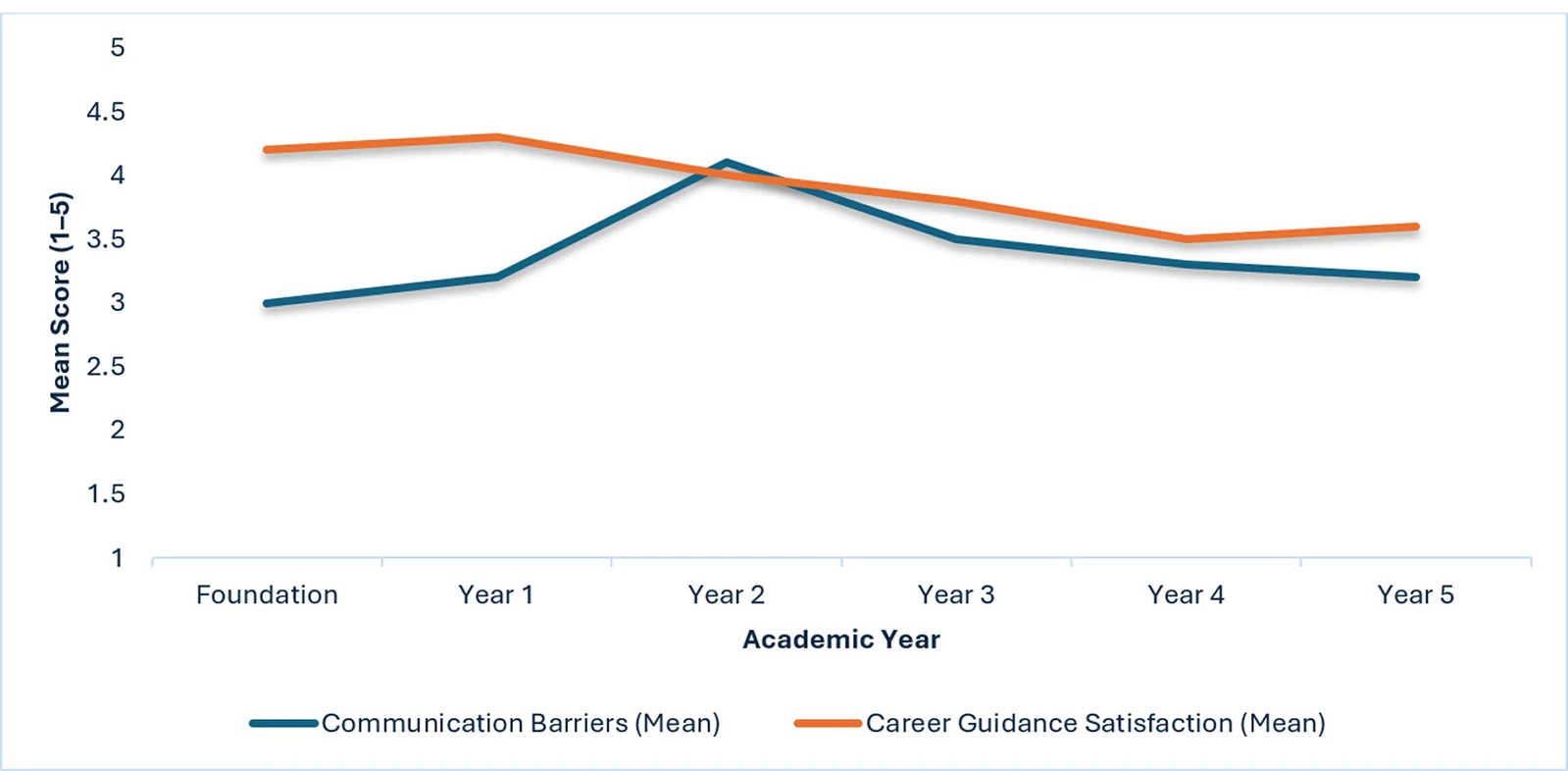
Cross-sectional variation in mentorship perceptions across academic years at DSMA. The chart displays differences in mentorship-domain scores across academic-year groups from Foundation Year through Year 5. Year 2 showed the highest observed communication-barrier level in the cross-sectional academic-year comparison, while career-guidance ratings varied descriptively across academic-year groups. These patterns informed subsequent qualitative exploration of mentorship experiences across training stages.

**Table 2.** ANOVA results across academic years at DSMA (Including effect sizes).

Mentorship domain	F (5, 245)	p-value	Partial η ^2^	Significant After Bonferroni (α = .0083)
Career Guidance	4.49	.001	.084	Yes
Communication Barriers	4.97	< .001	.092	Yes
Balancing Medical & Military Roles	3.16	.009	.061	No
Academic Support	1.65	.148	.033	No

Partial η ^2^ values between .06 and .10 indicate moderate educationally meaningful differences, suggesting that mentorship perceptions shift in practically relevant ways across training stages rather than reflecting trivial statistical variation.

Partial η ^2^ values of .084 and .092 indicate moderate effect sizes for career guidance and communication barriers, respectively. After applying Bonferroni correction (α = .0083), differences across academic years in balancing medical and military responsibilities did not meet the adjusted significance threshold (p = .009). Accordingly, the present quantitative analysis does not provide sufficient evidence of between-year variation in this domain; importantly, this non-significant finding should not be interpreted as evidence that levels of dual-role strain are equivalent across academic years.

Qualitative accounts nevertheless suggested that dual-role strain was experienced across different stages of training, although the present quantitative analysis cannot establish uniformity across academic years. Thus, while significant academic-year differences were detected for career guidance and communication barriers, the adjusted analysis did not provide sufficient evidence of academic-year differences in balancing medical and military responsibilities.

### Gender findings (exploratory)

Independent samples t-tests were conducted to explore gender differences within the DSMA cohort. A small statistically significant difference was observed in balancing medical and military responsibilities (p = .032), with male cadets reporting slightly higher perceived strain. No statistically significant gender differences were identified for academic support, career guidance, communication barriers, mentor availability, or career influence.

Given the small female subsample (n = 28), these findings should be interpreted as exploratory and hypothesis-generating (see Table 3).

**Table 3.** Gender comparison of key mentorship domains (DSMA).

Category	Male Mean (SD) (n=223)	Female Mean (SD) (n=28)	p-value
Academic support	3.96 (0.75)	3.79 (0.50)	.115
Career guidance	3.97 (0.68)	4.00 (0.27)	.660
Communication barriers	3.27 (1.10)	3.14 (0.89)	.478
Balancing dual roles	3.34 (0.91)	3.07 (0.90)	.032*

*Note:* Gender analyses were exploratory. Reported p-values were not adjusted for multiple comparisons and should not be interpreted as confirmatory evidence.

### Qualitative findings (DSMA only)

Thematic analysis of mentor interviews and cadet focus groups produced three themes that contextualized quantitative “friction points.”

### Hierarchical barriers

Cadets and mentors described rank differentials and authority gradients as inhibiting open communication. Hesitancy in initiating contact—linked to hierarchical distance and generational gaps—aligned with elevated quantitative communication barrier scores.

Participants emphasized how the uniform creates a psychological wall. One cadet noted: “I want to ask my mentor about feeling overwhelmed, but he is a Colonel. I am afraid he will see my struggle as a lack of military discipline rather than a medical student’s stress.” This illustrates reluctance to engage in psychosocial mentoring due to rank differentials.

This perception was also recognized by mentors. One mentor explained, ‘Mentees hesitate to approach mentors due to differences in rank and generational gaps’ (Mentor 6). Together, these accounts suggest that perceived hierarchical distance may constrain psychosocial accessibility and mentee-initiated communication.

This qualitative evidence explains the quantitative ‘friction point’ seen in the survey data, where rank acts as a persistent psychosocial moderator that limits mentee outreach.

### Dual-role strain

Cadets described the concurrent demands of medical coursework and military training as emotionally taxing and cognitively demanding. This theme was reported across academic years, supporting the interpretation that dual-role strain is systemic.

One mentee stated, ‘Balancing my academic responsibilities with military duties has been incredibly difficult. At one point, I even considered withdrawing’ (Mentee 8). A mentor similarly observed, ‘Many cadets struggle to balance training schedules with study time, and this is where mentors must step in’ (Mentor 5). These accounts indicate that competing medical and military responsibilities constituted an important contextual challenge for participants. However, consistent with the adjusted quantitative analysis, these qualitative observations should not be interpreted as evidence that levels of dual-role strain were statistically equivalent across academic years.

### Mentorship as identity integration

Mentors were perceived not only as academic advisors but also as exemplars of successful dual-role reconciliation. Cadets described mentors as symbolic bridges between clinical competence and military professionalism, helping explain why leadership development remained robust despite structural constraints.

Mentors acknowledged this bridging role. One mentor stated, “I show them that my stethoscope and my rank insignia are not in conflict; one informs the other. You cannot be a good military doctor if you forget how to be a doctor first.”

A mentee similarly described mentorship across both academic and military-developmental domains: ‘My mentor encouraged me to improve not only academically but also in discipline and leadership’ (Mentee 7). This account is consistent with mentorship extending across academic and military-role development, although it does not independently establish identity integration as a directly measured construct.

### Integrated interpretation

Integrated interpretation indicates that institutional hierarchy shapes mentorship accessibility—particularly psychosocial openness—while mentorship benefits related to career guidance and leadership remain comparatively robust. Together, the findings inform the Dual-Identity Dilemma as a conceptual interpretive framework. The quantitative component identified structural and relational friction points, whereas identity-related interpretation emerged principally from the qualitative findings and theoretical integration ( Kram, 1988; Zachary & Fain, 2022).

## IV. Discussion

This study demonstrates that mentorship is highly valued across both civilian and military medical education contexts; however, institutional structure shapes mentorship accessibility, particularly in the psychosocial domain. Using **AUSOM** as a civilian benchmark, **DSMA** cadets reported comparatively greater communication barriers and persistent strain associated with simultaneously meeting academic and military demands. The “Mentorship Paradox” identified at DSMA—characterized by high communication barriers (65.2%) despite high perceived value—contrasts sharply with the civilian model observed at Avalon. At AUSOM, where communication barriers were reported as minimal, the mentoring relationship is heavily focused on academic support and residency matching—an objective evidenced by their 92% first-time applicants’ residency (postgraduation placement) match success rate in 2025. Furthermore, while satisfaction in civilian contexts like Avalon is often driven by institutional success markers such as program surveys required by accreditation agencies, quality improvements, and student satisfaction rates ( Arja et al., 2025), the military context at DSMA necessitates a mentorship model focused on role reconciliation and resilience amid hierarchical dual-role strain.

We therefore use the term ‘Mentorship Paradox,’ previously described in the institution-focused DSMA analysis ( Phyo et al., 2025a), to characterize the coexistence within DSMA of high perceived mentorship value and substantial communication barriers, rather than as a phenomenon established through causal comparison with AUSOM.

### The Dual-Identity Dilemma as a structural condition

The Dual-Identity Dilemma represents tension between professional identity formation and institutional role conformity. In civilian contexts, mentorship appears primarily organized around academic coaching and career progression. In contrast, within military settings, mentorship must operate within hierarchical structures that may limit relational openness and constrain psychologically safe dialogue ( Griffin, 2017; Zachary & Fain, 2022).

These contrasts are descriptive and should not be attributed solely to the military–civilian distinction. Within DSMA, hierarchical structures may nevertheless influence relational openness and psychologically safe dialogue ( Griffin, 2017; Zachary & Fain, 2022). These qualitative accounts are consistent with the possibility that dual-role demands involve not only competing tasks but also tensions in how cadets reconcile medical and military role expectations. In this high-stakes environment, the qualitative findings suggest that mentors may function as ‘identity integrators’ by modelling ways in which clinical and military role expectations can coexist. Structural consistency and flexibility with mentees are well recognized in literature as key elements of the frameworks involving mentor-mentee relationships ( Tan et al., 2018).

Consistent with Social Learning Theory, developmental confidence is shaped by accessible role modeling and supportive interactions ( Bandura & Walters, 1977). When authority gradients discourage help-seeking or candid disclosure, mentees may experience reduced confidence in initiating developmental conversations. In this way, hierarchy operates not merely as an administrative feature but as a psychosocial moderator of mentorship effectiveness. Thus, the Dual-Identity Dilemma extends beyond competing tasks and reflects a deeper tension between competing professional selves—being socialized simultaneously into the values of a compassionate healer and a disciplined military officer. In this high-stakes environment, the mentor must act as an “identity integrator,” demonstrating that reconciling these dual roles is not only a practical requirement but also a hallmark of professional excellence ( Phyo et al., 2025b).

### Mentoring functions under the hierarchy

Kram’s (1988) framework distinguishes career functions (e.g., sponsorship, coaching) from psychosocial functions (e.g., counselling, affirmation). In DSMA, career guidance remained relatively strong overall, yet communication barriers suggest that psychosocial mentoring may be more difficult to access when relational approachability is constrained. This aligns with evidence that psychological safety is a key prerequisite for effective mentoring relationships ( Zachary & Fain, 2022).

### Developmental timing across academic years

Within DSMA, perceptions were not uniform across academic years. Year 4 respondents reported lower descriptive career-guidance ratings than several earlier-year groups, while Year 2 respondents showed the highest descriptive communication-barrier level. These cross-sectional patterns may indicate stage-related differences in mentorship needs, but they should not be interpreted as within-person developmental change ( Pitts, 2009). Longitudinal research is needed to determine how mentorship experiences evolve as individual cadets progress through training. These findings support the need for stage-sensitive mentorship structures and structured support during transition points ( Pitts, 2009).

### Mentorship as identity integration

Qualitative findings extend the quantitative results by indicating that mentors may function as “identity integrators”—models of how clinical professionalism can coexist with military expectations. This integrative function may help explain why leadership development remained robust despite structural barriers. In dual-role institutions, mentorship may therefore serve not only as academic support but as a mechanism of identity reconciliation ( Kram & Hall, 1989).

By functioning as identity integrators, mentors provide observational learning, central to Social Learning Theory, demonstrating that the reconciliation of dual identities is achievable and may serve as a marker of excellence in this setting ( Bandura & Walters, 1977). However, identity conflict and identity integration were not directly measured as quantitative constructs in the present study. Accordingly, the “identity integrator” interpretation should be regarded as a qualitative and hypothesis-generating interpretation rather than as an empirically validated mechanism.

The AUSOM cohort provides a useful descriptive civilian benchmark for contextualizing these findings, but it should not be interpreted as representing an identity structure directly comparable with that of DSMA. Previous exploratory work by members of our research group described related concepts of a ‘Paradox of Resilience’ and a ‘Strategic Adaptation Toolkit’ in military medical trainees ( Phyo et al., 2025b). These concepts have not yet been independently validated and are therefore referenced here only as contextual interpretive constructs rather than as established theoretical mechanisms. The present findings do not demonstrate that mentorship resolves dual-identity conflict; instead, they suggest that mentors may provide observational examples and psychosocial support that assist cadets in navigating potentially competing clinical and military role expectations. Future studies should directly measure professional identity formation and identity conflict to test the proposed mechanism.

While the civilian model at AUSOM focuses on a singular professional identity, the military mentor at DSMA must perform a “dual-modeling” role, demonstrating that the values of a “healer”—such as empathy and patient advocacy—can coexist within the rigid norms of an “officer” without compromising either identity. This demonstrates to mentees that reconciling dual identities is not only a practical requirement but also a hallmark of professional excellence in the military-medical setting ( Phyo et al., 2025b). Furthermore, while civilian mentorship satisfaction often aligns with external accreditation milestones ( Arja et al., 2025), the military mentor serves as the primary internal vehicle for navigating this complex identity.

### Practical implications

The findings suggest that mentorship models in dual-role training environments cannot rely solely on conventional structures designed for civilian medical education. Although DSMA cadets reported substantial benefits (career guidance and leadership development), the most prominent barriers—communication difficulty and persistent dual-role strain—appear embedded in hierarchical constraints. Where mentoring is formally assigned, but psychologically constrained, psychosocial mentoring functions may be reduced ( Zachary & Fain, 2022).

Accordingly, DSMA may benefit from a military-adapted mentorship approach that increases relational accessibility without undermining institutional discipline. Based on Social Learning Theory, developmental confidence is strengthened through approachable role modelling and supportive interaction ( Bandura & Walters, 1977). As proposed in the Military Mentorship Adaptation Model (see Figure 3), a tiered structure allows for the formal preservation of military hierarchy while creating a ‘safe’ base for psychosocial development through near-peer interactions. Strategies aligned with the present findings include:

1. Near-peer mentoring (e.g., Year 5 → Year 2) to reduce approachability barriers while maintaining organizational structure.
2. Protected mentorship time to reduce “mentoring as extra duty” perceptions and improve availability.
3. Mentor development training emphasizing psychosocial mentoring skills (listening, feedback, trust-building) alongside academic coaching, consistent with Kram’s (1988) distinction between career and psychosocial functions.
4. Flexible communication pathways, including asynchronous options, to reduce authority-related barriers while preserving organizational norms ( Zachary & Fain, 2022).

**Figure 3.**
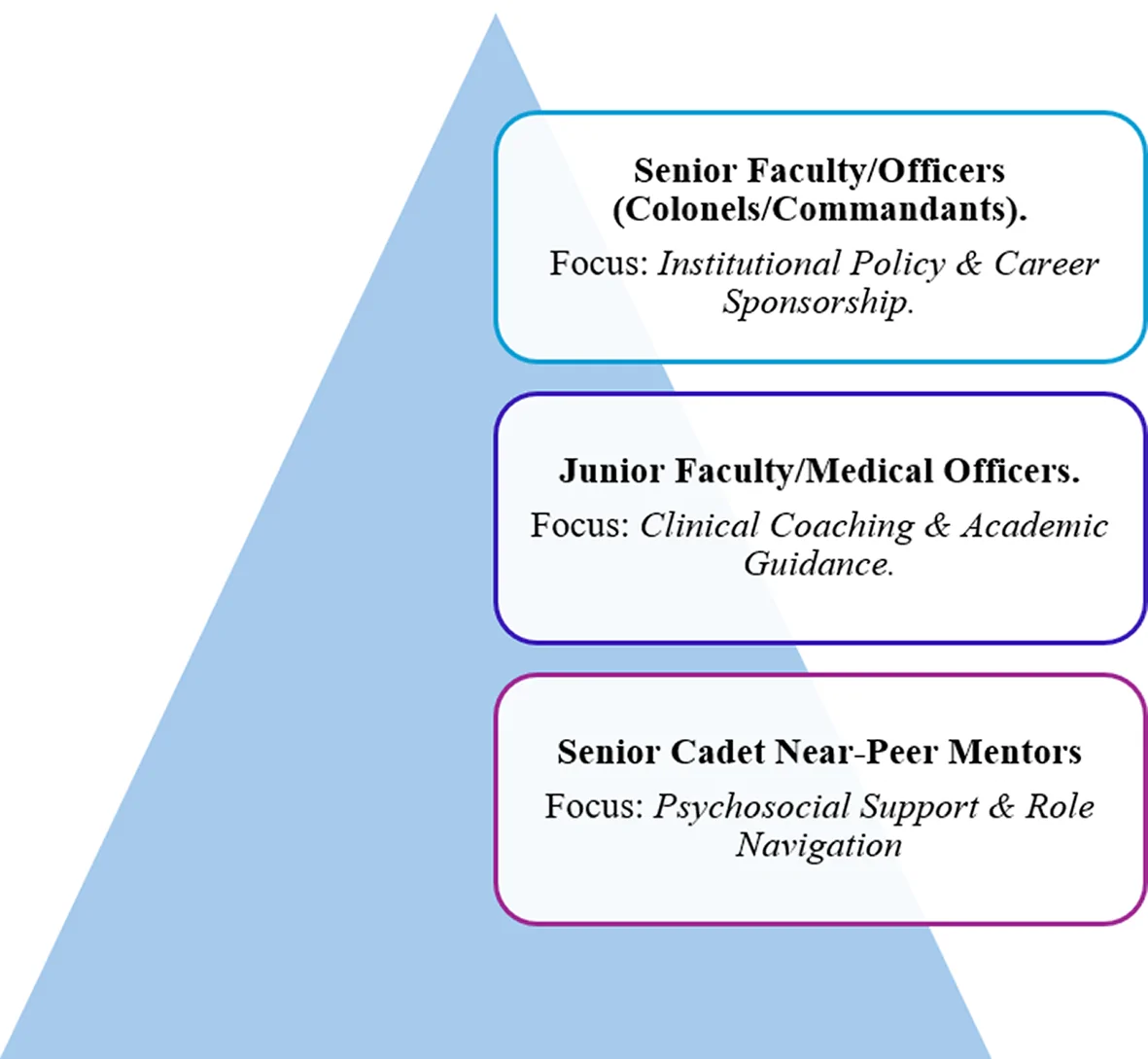
Military Mentorship Adaptation Model (proposed). This hypothesis-generating framework was developed from the integrated study findings and relevant literature. It was not implemented or empirically evaluated as an intervention in the present study. Future prospective evaluation is required to determine its feasibility and effectiveness.

The proposed Military Mentorship Adaptation Model ( Figure 3) should therefore be regarded as a hypothesis-generating framework derived from the integrated findings rather than as an empirically validated intervention. Its feasibility, acceptability, and effectiveness require prospective evaluation before implementation can be recommended.

### Strengths and limitations

Strengths include the near-census DSMA response rate and the explanatory sequential mixed-methods design, which enabled qualitative contextualization of quantitative patterns ( Creswell & Clark, 2023). Limitations include descriptive (non-causal) cross-institutional comparison, absence of qualitative data from AUSOM, and limited female representation.

Several limitations should be considered when interpreting these findings. First, the comparison between DSMA and AUSOM is descriptive rather than causal. The institutions differ substantially in mission. These contextual differences introduce potential confounding variables that prevent attribution of observed differences solely to the military–civilian distinction. Accordingly, AUSOM is best interpreted as a contextual benchmark rather than an experimental control group.

Second, qualitative inquiry was conducted only at DSMA. Explanatory mechanisms (“why”) are therefore grounded in the military context alone, while civilian findings remain limited to survey description. Including qualitative inquiry in future research at AUSOM would strengthen comparative interpretation.

Third, although moderate effect sizes (partial η ^2^) were reported for key ANOVA findings, future studies should strengthen interpretation by reporting confidence intervals and additional measures of practical significance. In large samples, statistical significance may not reflect practical significance. Future reporting should include effect-size estimates (e.g., partial η ^2^) alongside ANOVA outcomes to enhance interpretability.

Fourth, the gender analysis is exploratory due to the small female subsample. Limited statistical power may obscure meaningful differences. As female cadet representation increases, future studies should examine whether mentorship experiences differ by gender, particularly with respect to psychological safety and identity negotiation.

Fifth, survey administration also differed between institutions: DSMA participants completed paper-based surveys within the academy, whereas AUSOM participants completed an online SurveyMonkey survey. Together with the substantially different response rates (98.4% and 50%, respectively), these differences may have introduced mode-related, selection, and social-desirability biases. Institutional hierarchy and researcher affiliation may also have influenced participants’ willingness to disclose difficulties. The direction and magnitude of these potential biases cannot be determined.

Finally, although the manuscript conceptualizes a Dual-Identity Dilemma, quantitative measures primarily captured dual-role strain (balancing responsibilities) rather than identity-level conflict. While qualitative themes suggest possible identity integration through role modelling, future studies should incorporate direct measures of professional identity formation, such as the scale developed by Tagawa (2019), together with identity-focused qualitative prompts, to strengthen construct alignment and directly test the proposed Dual-Identity Dilemma mechanism.

## V. Conclusion

Mentorship in military medical education operates within structural conditions that differ meaningfully from civilian contexts. While mentorship remains highly valued across both settings, hierarchical authority gradients and dual-role demands shape its accessibility and the depth of its psychosocial impact within military institutions.

This study uses the Dual-Identity Dilemma as a conceptual lens for understanding structural tension between medical and military role expectations. Quantitative findings identified mentorship barriers and dual-role strain, while qualitative findings suggested that mentors may contribute to identity reconciliation through role modelling. Because identity conflict itself was not directly measured quantitatively, these identity-related interpretations should be considered hypothesis-generating and warrant direct evaluation in future research.

Dual-role institutions may therefore consider developing and prospectively evaluating structurally responsive mentorship approaches that acknowledge hierarchy while fostering relational accessibility. Tiered, developmentally sensitive mentorship structures represent one hypothesis-generating approach that warrants evaluation for feasibility, acceptability, and effectiveness before broader implementation.

## Data Availability

The data supporting this study’s findings are openly available in the Figshare repository at: https://doi.org/10.6084/m9.figshare.31388209 ( Phyo et al., 2026). Phyo, Z., Aung, Y. P., & Fatteh, R. A. (2026). The Dual-Identity Dilemma: Mentorship Structural Barriers Dataset. *Figshare.* https://doi.org/10.6084/m9.figshare.31388209
• License Data are available under the terms of the Creative Commons Attribution 4.0 International license (CC-BY 4.0).
