# Supplementary material for: The Dual-Identity Dilemma: A Comparative Study of Mentorship Structural Barriers in Military and Civilian Medical Education
Source: MedEdPublish (2016). 2026 Sep 12;16:19. Originally published 2026 May 26. [Version 2] doi: 10.12688/mep.21548.2 (PMC13646550; doi:10.12688/mep.21548.2)
Supplement: Peer review history

# Reviewer report: Reviewer response for version 2

**DOI:** 10.21956/mep.23401.r46671

**Published:** 2026-10-07

**Authors:** Anila Jaleel

**Affiliations:** 1 Shalamar Medical and Dental College, Lahore, Pakistan

The authors have incorporated the suggestions adequately. I recommend it to be accepted for indexing

Have any limitations of the research been acknowledged?

Yes

Is the work clearly and accurately presented and does it cite the current literature?

Yes

If applicable, is the statistical analysis and its interpretation appropriate?

Yes

Are all the source data underlying the results available to ensure full reproducibility?

Yes

Is the study design appropriate and is the work technically sound?

Yes

Are the conclusions drawn adequately supported by the results?

Yes

Are sufficient details of methods and analysis provided to allow replication by others?

Yes

Reviewer Expertise:

NA

I confirm that I have read this submission and believe that I have an appropriate level of expertise to confirm that it is of an acceptable scientific standard.

# Reviewer report: Reviewer response for version 1

**DOI:** 10.21956/mep.23086.r46425

**Published:** 2026-08-19

**Authors:** Anila Jaleel

**Affiliations:** 1 Shalamar Medical and Dental College, Lahore, Pakistan

This is a reasonably well-conceived explanatory sequential mixed-methods study with a genuinely interesting conceptual contribution (the Dual-Identity Dilemma framework), but it has several methodological vulnerabilities. Below is a structured appraisal.

- The authors are appropriately cautious in labeling AUSOM a "descriptive benchmark" rather than a control group, and they repeat this caveat several times.
- The abstract, results, and discussion all lean on the AUSOM-vs-DSMA contrast as the central evidentiary pillar for the "Mentorship Paradox," even though the two institutions differ in curriculum length (4-year MD vs 6-year integrated), national context, survey delivery mode (paper/verbal consent vs SurveyMonkey/written consent), response rate (98.4% vs 50%), and career stage composition.
- The paper's rhetorical structure reads as causal even though the numbers are purely descriptive. This is a common technical debt in comparative-benchmark designs. 2. Statistical treatment of Likert data
- The authors never justify the choice or check normality/homogeneity of variance as no Levene's test reported, no rationale for treating ordinal data as interval.

- Balancing Medical & Military Roles (p = .009) is called *not* significant after Bonferroni (α = .0083), which is correct arithmetically, yet the authors then argue this null result "indicates that dual-role strain is a pervasive structural challenge rather than a developmentally specific phenomenon" i.e., interpreting a non-significant ANOVA as evidence of *uniformity* rather than absence of evidence. That's a classic absence-of-evidence as evidence of absence error, a null ANOVA across years tells the variation-by year wasn't detected, not that the underlying construct is pervasive.

- To their credit, the authors flag the n = 28 female subsample as exploratory and non-Bonferroni-adjusted.  But the single significant result (dual-role balancing, p = .032, uncorrected) is reported in the abstract adjacent narrative without the same hedging it receives in the limitations. Readers skimming Table 3 could easily  treat this as a robust finding.

 Qualitative component

- Only 8 mentor interviews + 2 focus groups (n = 8 mentees) at a single site, with no explicit statement of saturation, no inter-coder reliability/kappa reported despite using MAXQDA, and no researcher-reflexivity statement given that some authors are embedded. DSMA faculty interviewing their own cadets/mentors is a real risk of social desirability bias in a hierarchical military setting where cadets are being asked to candidly discuss authority-related discomfort with people connected to their own institution.
- The two illustrative quotes are vivid and support the hierarchical wall theme well, but two quotes total across 10 qualitative encounters is thin evidentiary support for the thematic claims being built on top of them (three named themes).

- The paper's own limitations section admits the sharpest problem: the "Dual-Identity Dilemma" is framed throughout as an *identity-formation* construct, but the quantitative instrument actually measures *dual-role strain/balancing responsibilities*, a related but distinct construct (task/role conflict vs identity conflict). This is a meaningful construct-validity gap that undercuts the headline framing. The paper is more accurately titled/pitched as a study of structural/hierarchical barriers to mentorship, with identity integration emerging as a *qualitative* theme rather than something quantitatively measured.
- Figure 1's conceptual model and Figure 3's proposed tiered mentorship model are both plausible and pedagogically useful, but neither is empirically tested in this study. They're proposed based on the qualitative findings plus prior literature, which is fine as long as they're presented as hypothesis-generating .However Figure 3 is captioned in a way that could be mistaken for a validated intervention).

- Several of the paper's most theoretically load-bearing claims (the "Paradox of Resilience," "Strategic Adaptation Toolkit," "identity integrator" framing) are attributed to Phyo et al. 2025 ,an earlier paper by the same lead author. This isn't disqualifying, but it means the conceptual scaffolding rests heavily on the authors' own prior, largely unreplicated framework rather than external validation, which needs to be contextualized more explicitly.

- Verbal consent (DSMA) vs written/electronic consent (AUSOM) is disclosed transparently, which is good, but combined with the participation format difference (paper-based/mandatory-feeling institutional survey vs voluntary online),  raises a subtle concern about response-bias asymmetry that compounds the benchmark comparability problem above. Cadets responding to a paper survey administered within a military academy structure may feel differently able to report communication barriers freely than the framing assumes, in either direction

**MINOR ISSUES:**

- Author affiliation line has a typo ("Curaçaoad,,").
- The introduction repeats the aims/RQ paragraph almost verbatim (once before Figure 1, once after).
- "Kram, 1988" is cited in-text but the reference list gives it as *Kram KE: Mentoring at work…1988*, consistent , but earlier in-text citations alternate between "Kram & Hall, 1989" and "Kram, 1988"..

**Final comments: Major Revision is required**

**Acknowledgement: I have used Claude AI to assist in Peer review of this article**

Have any limitations of the research been acknowledged?

Yes

Is the work clearly and accurately presented and does it cite the current literature?

Yes

If applicable, is the statistical analysis and its interpretation appropriate?

Yes

Are all the source data underlying the results available to ensure full reproducibility?

Yes

Is the study design appropriate and is the work technically sound?

Yes

Are the conclusions drawn adequately supported by the results?

Yes

Are sufficient details of methods and analysis provided to allow replication by others?

Yes

Reviewer Expertise:

Biochemistry and molecular biology, medical education and digital technology in Medical education

I confirm that I have read this submission and believe that I have an appropriate level of expertise to confirm that it is of an acceptable scientific standard, however I have significant reservations, as outlined above.

# Response

**Published:** 2026-08-25

**Authors:** Zaw Phyo

**Affiliations:** Medical Education Department, Not Applicable, Yangon, Yangon, Myanmar

We sincerely thank the reviewer for the careful and constructive evaluation of our manuscript. The comments were valuable in improving the methodological transparency, interpretation, and presentation of the study. We have revised the manuscript accordingly and provide our point-by-point responses below.  

**1. AUSOM as a descriptive benchmark and risk of causal interpretation** We agree. The revised manuscript consistently describes AUSOM as a **descriptive contextual benchmark rather than a causal control group**. We have strengthened this clarification in the Results, Discussion, and Limitations and explicitly acknowledge differences in curriculum, institutional and national context, learner composition, survey administration, and response rate. We also clarify that the observed between-institution differences cannot be attributed specifically to military hierarchy. This clarification is now explicit in the comparative Results and Discussion.  

**2. Statistical treatment of Likert data and assumption testing** We agree that the statistical approach required clearer reporting. The revised Methods now provide a rationale for the use of parametric analyses with Likert-type data, supported by Norman (2010), and describe assessment of distributional and variance assumptions using the Shapiro–Wilk and Levene tests. The manuscript also specifies the use of Welch-adjusted analyses where appropriate. These additions are included in the revised Data Analysis section.

**3. Interpretation of the non-significant finding for balancing medical and military roles** We agree. We have removed the previous interpretation that non-significance demonstrated uniformity across academic years. The revised manuscript now states that the analysis provides insufficient evidence of between-year variation and explicitly notes that a non-significant result should **not** be interpreted as evidence of equivalence across academic years. The revised interpretation appears in the Results section.  

**4. Exploratory gender finding** We agree. The gender finding has been removed from the Abstract and is now clearly identified in the Results and Table 3 as **exploratory, unadjusted, and hypothesis-generating rather than confirmatory**. We also emphasize the limited female representation in the Limitations section. The revised treatment is shown in the gender Results and table note.  

**5. Qualitative sampling, saturation, coding, reflexivity, and hierarchical response bias** We agree. The qualitative Methods have been expanded substantially. The revised manuscript now describes purposive sampling, thematic saturation, private data-collection settings, use of non-supervisory faculty, avoidance of direct supervisory or hierarchical interviewer–participant relationships, field notes, consensus-based coding, an audit trail, analyst triangulation, reflexive memoing, examination of discrepant cases, and researcher positionality. Coding agreement was addressed through consensus-based analytic review rather than calculation of a formal kappa coefficient, and this is now stated explicitly. These revisions are presented in the Qualitative Phase and Data Analysis sections.  

**6. Limited number of illustrative quotations** We agree. The revised Results now include additional illustrative quotations from both mentors and mentees across the three qualitative themes: hierarchical barriers, dual-role strain, and mentorship as identity integration. These additional quotations are drawn from the original qualitative dataset; no new qualitative data were collected for the revision. The expanded quotations are presented in the revised qualitative Results.  

**7. Construct validity of the Dual-Identity Dilemma**. We agree with this important distinction. The revised manuscript now presents the **Dual-Identity Dilemma as a conceptual interpretive framework**, rather than as a quantitatively validated identity construct. We distinguish the quantitative assessment of structural barriers and dual-role strain from identity-related interpretations that emerged principally from the qualitative findings and theoretical integration. We also explicitly acknowledge in the Limitations that identity conflict and identity integration were not directly measured quantitatively and recommend direct professional-identity measures in future studies. These revisions are reflected in the Discussion and Limitations.  

**8. Figures 1 and 3 were not empirically tested** We agree. Both figures and their legends have been revised to make their status explicit. Figure 1 is now described as a **hypothesis-generating conceptual framework whose proposed relationships were not directly tested**, while Figure 3 is clearly labelled a **proposed, hypothesis-generating framework** that was not implemented or empirically evaluated as an intervention. The manuscript further states that prospective evaluation is required before implementation can be recommended.  

**9. Cross-sectional academic-year interpretation** We have also revised the academic-year interpretation and Figure 2 to emphasize the **cross-sectional** nature of the data. Year 2 is now described only as showing the highest observed communication-barrier level, without interpreting this as a longitudinal transition or within-person developmental trajectory. The Discussion explicitly states that these patterns should not be interpreted as within-person developmental change.  

**10. Reliance on prior work by Phyo et al.**. We agree that these concepts require appropriate contextualization. The revised manuscript now explicitly identifies the previously reported DSMA dataset and distinguishes the present comparative and conceptual analysis from the earlier institution-focused study. The “Paradox of Resilience” and “Strategic Adaptation Toolkit” are now explicitly described as **exploratory concepts that have not yet been independently validated**. They are used only as contextual interpretive constructs, while the present interpretation is additionally grounded in established literature on mentoring functions, observational learning, psychological safety, and professional identity formation. These clarifications appear in the Methods and Discussion.  

**11. Survey-mode and response-bias asymmetry** We agree. The revised Methods now describe the safeguards used at DSMA, including voluntary and anonymous participation, distribution and collection by non-teaching staff, sealed-envelope return, and attention to culturally appropriate and rank-sensitive wording. The revised Limitations also explicitly acknowledge potential mode-related, selection, social-desirability, and authority-related response biases associated with the differing survey procedures and response rates. We further state that the direction and magnitude of these potential biases cannot be determined from the present data. These revisions appear in the Methods and Limitations.  

**12. Consent procedures** We have reviewed and harmonized the consent descriptions throughout the revised manuscript. Informed verbal consent was obtained from DSMA participants before both quantitative and qualitative participation, while AUSOM participants provided electronic informed consent before beginning the online survey. Participation was voluntary in both institutions. The consent information is now consistent across the Ethical Considerations and Informed Consent sections.  

**13. Typographical error in the affiliation**

 Thank you. This typographical error has been corrected to **“Willemstad, Curaçao.”**  

**14. Repetition of the aims and research questions** Thank you. The duplicated material has been removed, and the revised manuscript now contains a single Study Aims and Research Questions section.  

**15. Kram citation consistency** Thank you. The mentoring-framework citations have been harmonized to **Kram (1988)**, consistent with the reference-list entry used throughout the revised manuscript.  

**Final response** We thank the reviewer again for the detailed and constructive critique. The revisions have helped us improve the transparency of the study design, strengthen reporting of the quantitative and qualitative methods, reduce causal and developmental overinterpretation, clarify the distinction between measured dual-role strain and identity-related interpretation, and more clearly position the Dual-Identity Dilemma and proposed mentorship model as hypothesis-generating frameworks requiring further empirical evaluation.

# Reviewer report: Reviewer response for version 1

**DOI:** 10.21956/mep.23086.r46301

**Published:** 2026-08-18

**Authors:** Devi Prasad Mohapatra

**Affiliations:** 1 Jawaharlal Institute of Postgraduate Medical Education & Research (JIPMER), Puducherry, India

In medical domain, professional identity is the internal sense of what one views self as a healthcare professional. It is shaped through knowledge, values, behaviours, and the norms of the professional community through socialization in classrooms, clinical environments, and interactions with mentors, peers, and patients. Professional identity guides ethical behaviour, supports resilience, also fosters belonging, and confidence. ^1, 2^

 The dual‑identity dilemma is a situation that arises when a learner must form two identities at the same time, each having different expectations, values, and behavioural norms. This dilemma may affect professional identity formation of an individual by slowing or complicating the process of internalizing the norms of the profession. Mentors play a crucial role in reconciling the identity dilemma and creating confident professionals with a secure professional identity.

 This is an interesting manuscript which aims to examine how mentorship functions within a dual‑identity training environment where medical cadets must develop both a professional medical identity and a military institutional identity. The authors have attempted to identify structural barriers that shape mentorship accessibility in a hierarchical setting of a defence school of medicine. Mentorship perceptions between the participants of a military academy and a civilian medical school have been compared to understand the role of institutional structure on communication, psychosocial safety, and professional identity formation.

 I find this manuscript idea novel and there isn’t much literature available conveying  a similar perspective. A sequential mixed‑methods design was employed by the authors. Quantitative survey data from defense medical academy cadets and civilian medical school students were collected to map mentorship expectations, satisfaction, and communication barriers. They then conducted qualitative interviews with participants from the defence medical academy group to explain the friction points which had been identified earlier in the quantitative phase.

 According to the authors, while mentorship is valued in both settings, it appears to be structurally constrained in military academic environments. They highlight the Dual‑Identity Dilemma, where cadets need to balance expectations of empathy‑driven medical professionalism versus rank‑based military discipline. The authors have suggested that authority gradients are perceived to reduce psychological safety and limit open communication, particularly during early transitional years. The study seems to show that mentorship challenges noticed in defense military academies may not be inherent to the medical education but rather to the hierarchical structure of the institution. The authors have clearly highlighted the limitations of their study, particularly the descriptive nature of the comparison between the two academies with differences in mission, culture, and curriculum. Qualitative data has also been collected only from the defense academic group and not from the civilian cohort, which may limit the depth of interpretation.

 Authors may consider measuring professional identity formation among participants in future studies which is missing in the present study, even though they have considered professional identity as central to the dual identity dilemma. Tools like Professional Identity Formation (PIF) scale may be used.3, 4

Have any limitations of the research been acknowledged?

Yes

Is the work clearly and accurately presented and does it cite the current literature?

Yes

If applicable, is the statistical analysis and its interpretation appropriate?

Yes

Are all the source data underlying the results available to ensure full reproducibility?

Yes

Is the study design appropriate and is the work technically sound?

Yes

Are the conclusions drawn adequately supported by the results?

Yes

Are sufficient details of methods and analysis provided to allow replication by others?

Yes

Reviewer Expertise:

Mentorship, medical education.

I confirm that I have read this submission and believe that I have an appropriate level of expertise to confirm that it is of an acceptable scientific standard.

## References

[1] : Belonging in dual roles: exploring professional identity formation among disabled healthcare students and clinicians. *Advances in Health Sciences Education* .2025;30(4) : 10.1007/s10459-024-10386-41101-1121 10.1007/s10459-024-10386-4 PMC1239086239509065

[2] : An interprofessional socialization framework for developing an interprofessional identity among health professions students. *Journal of Interprofessional Care* .2013;27(6) : 10.3109/13561820.2013.804042448-453 10.3109/13561820.2013.804042 23777592

[3] : Development of a scale to evaluate medical professional identity formation. *BMC Medical Education* .2019;19(1) : 10.1186/s12909-019-1499-9 10.1186/s12909-019-1499-9 PMC639402730819174

[4] : Developing questionnaires for educational research: AMEE Guide No. 87. *Medical Teacher* .2014;36(6) : 10.3109/0142159X.2014.889814463-474 10.3109/0142159X.2014.889814 PMC405919224661014

# Response

**Published:** 2026-08-18

**Authors:** Zaw Phyo

**Affiliations:** Medical Education Department, Not Applicable, Yangon, Yangon, Myanmar

​Dear Reviewer, ​Thank you very much for reading our paper and giving us such positive, helpful feedback. We are glad you found our research on the Dual-Identity Dilemma novel and interesting. ​Here is our response to your points:

- ​ **Praise and Summary:** Thank you for agreeing with our findings on how military rank can make open communication harder for cadets.

 

- ​ **Measuring Identity Formation in Future Studies:** We agree completely with your advice. In this paper, we focused on measuring communication problems and workload balance. In our next projects, we will use validated tools like the **Tagawa (2019) Professional Identity Formation (PIF) scale** to measure cadets' professional identity directly.

 

- ​ **Recommended Literature:** Thank you for sharing the articles by **Mayer et al. (2025)** and **Khalili et al. (2013)**. They are very useful for our future work.

  ​Thank you again for helping us improve our research. ​Sincerely, **Zaw Phyo** *(on behalf of all co-authors)* Corresponding Author, DSMA
